# Prevalence of unmet health care need in older adults in 83 countries: measuring progressing towards universal health coverage in the context of global population ageing

**DOI:** 10.1186/s12963-023-00308-8

**Published:** 2023-09-15

**Authors:** Paul Kowal, Barbara Corso, Kanya Anindya, Flavia C. D. Andrade, Thanh Long Giang, Maria Teresa Calzada Guitierrez, Wiraporn Pothisiri, Nekehia T. Quashie, Herney Alonso Rengifo Reina, Megumi Rosenberg, Andy Towers, Paolo Miguel Manalang Vicerra, Nadia Minicuci, Nawi Ng, Julie Byles

**Affiliations:** 1 International Health Transitions, Canberra, Australia; 2 Health Data Analytics Team, The Australian National University, Canberra, Australia; 3 Neuroscience Institute, National Research Council (CNR), Padua, Italy; 4 School of Public Health and Community Medicine, University of Gothenburg, Gothenburg, Sweden; 5 School of Social Work, University of Illinois at Urbana-Champaign, Urbana-Champaign, USA; 6 Faculty of Economics, National Economics University, Hanoi, Viet Nam; 7 Universidad del Valle, Cali, Colombia; 8 College of Population Studies, Chulalongkorn University, Bangkok, Thailand; 9 Department of Health Studies, University of Rhode Island, Kingston, USA; 10 Facultad de Odontología, Universidad El Bosque, Bogota, Colombia; 11 WHO Center for Health Development, Kobe, Japan; 12 School of Health Sciences, Massey University, Palmerston North, New Zealand; 13 Asian Demographic Research Institute, Shanghai University, Shanghai, China; 14 Department of Public Health and Community Medicine, University of Gothenberg, Gothenburg, Sweden; 15 School of Medicine and Public Health, University of Newcastle, Newcastle, Australia

**Keywords:** Health service needs and demand, Health services research, Adult, Older adult, Africa, Americas, Asia, Europe

## Abstract

Current measures for monitoring progress towards universal health coverage (UHC) do not adequately account for populations that do not have the same level of access to quality care services and/or financial protection to cover health expenses for when care is accessed. This gap in accounting for unmet health care needs may contribute to underutilization of needed services or widening inequalities. Asking people whether or not their needs for health care have been met, as part of a household survey, is a pragmatic way of capturing this information. This analysis examined responses to self-reported questions about unmet need asked as part of 17 health, social and economic surveys conducted between 2001 and 2019, representing 83 low-, middle- and high-income countries. Noting the large variation in questions and response categories, the results point to low levels (less than 2%) of unmet need reported in adults aged 60+ years in countries like Andorra, Qatar, Republic of Korea, Slovenia, Thailand and Viet Nam to rates of over 50% in Georgia, Haiti, Morocco, Rwanda, and Zimbabwe. While unique, these estimates are likely underestimates, and do not begin to address issues of poor quality of care as a barrier or contributing to unmet need in those who were able to access care. Monitoring progress towards UHC will need to incorporate estimates of unmet need if we are to reach universality and reduce health inequalities in older populations.

**Supplementary Information:**

The online version contains supplementary material available at 10.1186/s12963-023-00308-8.

## Introduction

The 1978 Alma Ata Declaration [1] identified health as a human right and ensuring access to health care as a primary responsibility of governments. These ground-breaking principles called for primary health care for all—with equity of access and affordability central to policies that support achieving universal health *care* [2]. Need was also included in the World Health Organization and UN Sustainable Development Goal (SDG) 3.8 definition of universal health *coverage* (UHC): “…people receive the health services they need without suffering financial hardship”. In the global context, need is not explicitly defined, nor does unmet need have an agreed standard definition. Quantifying the levels of unmet need contributes to efforts to “leave no one behind” as part of SDG Target 3.8 and renewed interest in equity in national policy deliberations as part of commitments to the Astana Declaration [3].

A number of definitions of health need are in use—with no agreed convention or standard definition. Care needs in adult populations are often inferred from rates of disease and disability, or administrative data on service utilization [4]. The International Labor Organization (ILO) recently defined need (for health care) related to older adults as any “*older persons aged at or above their healthy life expectancy*” [5]. Global disease estimates derived from entities like the Global Burden of Disease Study, indicate need for health care at a population level and allow comparisons between countries. These disease burden morbidity estimates can be used to forecast future health levels in different population groups or sub-groups over time, and the related health care requirements. Using this approach, need for health defined by disease burden is often invoked as a driver of demand on health care systems [6, 7].

Yet “need for health” at the population level defined using healthy life expectancy or disease burden, for example, likely differs from “health need” at the individual level, where health need is self-perceived and may include health issues for which there are no or limited effective treatment options [8]. Asking older persons whether or not their needs for health care have been met is a pragmatic way of capturing this information in this population group—and with additional follow-on questions, can also identify barriers in access to health services and service adequacy.

Direct questions about unmet need are asked as part of a number of health and social surveys worldwide, including a range of ageing and health studies. Based on responses to these questions, prevalence of self-reported unmet need can be estimated. For instance, some surveys ask a general question about unmet need, along the lines of “The last time you needed health care, did you get health care?”. Others include questions that seek to also identify the type of barriers people faced, for instance, cost of services, distance needed to travel to access the service, or waiting time at a facility. An example question would be, “During the past 12 months, was there a time when you had a medical problem but did not consult with/visit a doctor because of the cost?”.

Unmet need for health care is a concern for older individuals and societies (including loss of functioning or productivity from untreated health conditions), for national policymakers who aim to ensure older population health needs are met, and for global efforts to track progress toward UHC [9, 10]. For example, policies that alter financial levers to achieve UHC may not account for latent unmet need, leading to more inequities in demand and supply for older adult populations and those with multimorbidity or the most complex health concerns [11–13]. Summary indicators or indices developed to measure UHC might also need to account for unmet need [14].

Unmet need is already part of policy dialogue and data monitoring in European and OECD countries, where it is used as a measure of access to health care, and is derived from the data available through the EUROStat dashboard (see for example: https://ec.europa.eu/eurostat/web/products-datasets/-/tespm110). The European Commission included “self-reported unmet need for medical care” as one of 12 core indicators of its Social Scoreboard, and can be further disaggregated by older age groups and sex. The International Health Policy Survey carried out by the Commonwealth Fund in 11 industrialized countries includes direct questions on unmet need in populations aged 65 years and older (https://www.commonwealthfund.org/series/international-health-policy-surveys). Beyond Europe, countries asking respondents about unmet needs in national surveys include, for instance, Canada, Chile, New Zealand, Republic of Korea, Thailand, Turkey and the United States of America. Unfortunately, much of the published literature on the topic of unmet need is limited to higher income countries.

The aims of this paper were to generate the estimates of unmet health care need prevalence for adults aged 60 years and older (60+) in 83 countries in different world regions, and assess the distribution of unmet health care need by age, sex, and location where possible—with an eye to how the definitions used to generate these results would feed into measurement of university health coverage.

## Materials and methods

**Fig. 1:**
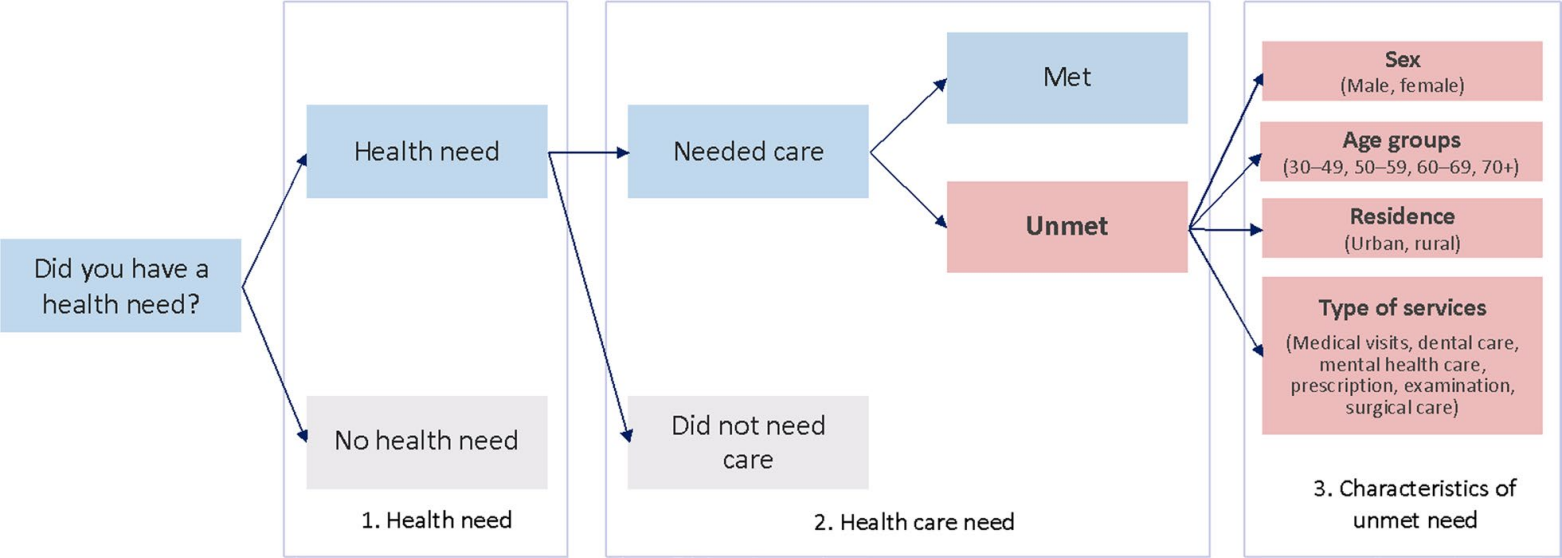
Decision tree for met and unmet health care need within selected surveys. *Notes*: Some of surveys started from the “health care need” (2) without asking respondents’ health need (1). A number of surveys asked about health need, then specifically asked whether or not the respondent felt she/he/they needed care for this health need (see Cambodia and The Gambia, for example). Others incorporated health need into the question about accessing care (SAGE, for example)

Questionnaires and study materials from 18 studies covering 83 countries across all world regions, were identified and reviewed. We had sought access to a wider range of studies, but could not include more of these because we are awaiting approvals for data access, language/translation issues, or the available variables did not allow us to proceed with analyses. The total sample size for each country is available in Additional file 1: Appendix 1. Questions about unmet health care need were evaluated for each study, and suitable questions were entered into a spreadsheet. This required assessment of differences in question wording, response categories and number of questions from a wide range of studies. Questions were compared and compiled across studies, with a decision tree used to define met and unmet need (Fig. 1). Questions were evaluated to construct the variables for analysis and to generate needed numerator and denominator information for each variable and each study (Table 1).

**Table 1:** Calculating unmet health care need—variable definitions used to generate prevalence estimates

*Did not need health care*
Definition: The proportion of respondents who reported that they did not need health care
*Met health care need*
Definition: The proportion of respondents who reported that their need for health care was met
*Unmet health care need*
Definition: The proportion of respondents who reported that they needed health care but did not receive it

In addition to the usual challenges of generating estimates from surveys with complex designs (including type of interview (phone, online, in-person), sampling, sample size, and question skip patterns), we expected variability in results between studies because of the nature of the question or questions asked about unmet need. The unmet need questions presented a number of additional challenges when looking across the different surveys, such as: single versus multiple questions; question wording; response categories; question flow and location within the interview; timeframes [open-ended versus time-specific (for instance, in last 30 days)]; whether a type (or types) of health care were specified (broadly, outpatient or inpatient care); and whether the questions specified a reason within the question itself (because of cost or accessibility), or if a follow-on question was asked that allowed the respondent to share the reason. A few salient examples are described below—with more question details available in Table 2 (see also, more detailed technical notes at https://extranet.who.int/kobe_centre/en/project-details/unmet_needs).

**Table 2:** Examples of unmet need questions by study

	Survey (year)	Question(s)
1	Brazilian longitudinal study of aging (ELSI-Brazil) (2015)	U32: Have you looked for a health service to get an appointment related to your health, in the PAST 2 weeks
		U34: Were you immediately taken care of on the first time you sought care in this health service in the PAST 2 weeks?
		U35: What was the reason for not being taken care of on the first time you sought care in this health service in the PAST 2 weeks?
		U36: In this latest appointment, within the PAST 2 Weeks, were you prescribed any medication?
		U37: Were you able to get all the medication that was prescribed to you on your latest appointment within the PAST 2 weeks?
		U38: Why didn’t you get all the medication that was prescribed to you on your latest appointment within the PAST 2 weeks?
		T6: In the PAST 30 days, due to financial issues, you:
		(1) Had no financial issues to buy the medication(s)
		(2) Did not take a medication that was prescribed by a doctor or a dentist
		(3) Decrease the number of pills of the medication(s) that were prescribed by the doctor
		(4) Decreased the dose of the medication, breaking the pills or taking less drops
		(5) Didn’t use medication(s)
		(9) Didn’t know/didn’t answer
2	Cambodia elderly survey (CES) (2004)	G20: During the past year, were there any times that you were sick or injured?
		G21: For how many days, if any, during the last year would you say you were unable to perform your usual activities because of these illnesses or injuries?
		G22: Did you receive any professional treatment or take any medicines for these illnesses or injuries over the past year?
		G23: Do you think that you needed such treatment or medicines?
		G24: What were the reasons that you did not receive this treatment?
3	Commonwealth fund international health policy survey (CMWF) (2017)	QN810: During the past 12 months, was there a time when you:
		qn810_a1: Did not fill/collect a prescription for medicine, or you skipped doses of your medicine because of the cost
		QN810_a2: Had a medical problem but did not consult with/visit a doctor because of the cost
		QN810_a3: Skipped a medical test, treatment, or follow-up that was recommended by a doctor because of the cost
		QN810_a4: Did not visit a dentist when you needed to because of the cost
4	Integrated household survey (IHS), The Gambia (2016)	S2aq3_1: During the last 2 weeks, what symptoms has [name] suffered from?
		S2aq4_1: Did [NAME] consult a health provider for this illness/injury last 2 weeks for MAIN illness?
		S2aq5_1: What was the main reason that [NAME] did not visit a health practitioner during his/her illness?
		S2aq3_2: During the last 2 weeks, what symptoms has [name] suffered from?
		S2aq4_2: Did [NAME] consult a health provider for this illness/injury last 2 weeks for SECONDARY illness?
5	Mexican health and aging study (MHAS) (2001–2018)	[2001 and 2003] D15: In the last 5 years, was there at least one instance when you had a serious health problem but you did not go to the doctor?
		[2012, 2015, 2018] d15_15: Last 2 years: Did respondent have a serious health problem but not seek a doctor?
6	Myanmar aging study (MAS) (2012)	J41: During the past 12 months, were there any times that you were sick or injured that prevented you from performing your usual activities?
		J42: Because of illnesses or injuries during the last 12 months, how many days were you unable to perform your usual activities because of these?
		J43: Did you receive any professional treatment for these illnesses or injuries?
		J44: Do you think that you needed treatment?
7	Myanmar survey on accessing healthcare to older persons (2016)	C1: During the past 12 months, were there any time that you were sick or injured and needed health care?
		C3: The last time you needed health care, did you get health care?
8	Study of global AGEing and adult health (SAGE) Mongolia (2018)	Q5002: The last time you needed health care, did you get health care?
9	New Zealand Health Survey (NZHS) (2015, 2016, 2017, 2018, 2019)	A2.06: In the past 12 months, has there been a time when you wanted to see a GP, nurse or other health care worker at your usual medical centre within the next 24 h, but they were unable to see you?
10	Puerto Rican Elderly: health conditions (PREHCO) (2003, 2007)	J1/WJ1 Could you tell me how many medications, prescribed by a doctor, you have been taking regularly in the last year?
		J12/WJ12 During the last year, have you stopped taking or have you taken less of any medication that was prescribed because you could not afford it?
		K48/WK48 In the last 12 months, were you ever told you should get an x-ray or have laboratory tests done, not including those for a hospitalization? K49/WK49 Did you have those tests done?
		K60/WK60 In the last 2 years, have you needed medical attention that you could not get?
11	SAGE wave 1 (Ghana, India, China, Mexico, Russia, South Africa) and Wave 2 (Ghana, Mexico, South Africa) (2007–2019)	Q5001: When was the last time that you needed health care?
		Q5002: The last time you needed health care, did you get health care?
		Q5003a. What was the main reason you needed care, even if you did not get care?
		Q4067: During the last 12 months, have you had any problems with your mouth and/or teeth, including problems with swallowing?
		Q4068a: Have you received any medications or treatment from a dentist or other oral health specialist during the last 2 weeks?
		Q4068b: Have you received any medications or treatment from a dentist or other oral health specialist during the last 12 months?
12	Survey on health, well-being, and aging (SABE) Colombia (2015)	P902: During the last 30 days, have you had any health problems?
		P903: For any of the health problems you have had in the last 30 days, have you consulted or sought help?
		P904: What was the main reason for NOT consulting or seeking help?
		P905: Did you receive the requested care from your health service for the problems that you have experienced?
	SABE Ecuador (2009)	E9: In the last year—did not take medication
		F3_2: In the last year—did not consult even if needed it
		F20aL: These exams (radiographies) were performed (for people who need medical attention)
		C.17 In the last year have you been cared for by a dentist?
13	Thai health and welfare survey (HWS) (2011, 2013, 2015, 2017)	UN1 (out-patient): Was there any time during the last 12 months when you were sick and needed a medical treatment but you did not receive it?
		UN3 (in-patient): Was there any time during the last 12 months when you needed or were recommended by medical doctor to admit to a health facility but you did not receive it?
14	Health examination survey (THES) (2016)	A5010: The last time you needed health care, did you get health care?
15	Vietnam national aging survey (VNAS) (2011)	I18: During the last 12 months, were there any times that you were sick or injured that prevented you from performing your usual activities?
		I20: Did you receive any professional treatment for these illnesses or injuries over the last 12 months?
		I21: Do you think that you needed treatment?
16	Survey on older persons and Social Health Insurance (OP&SHI) (2019)	Q0604: During the last 12 months, were there any times that you were sick or injured that prevented you from performing your usual activities?
		Q0606: Did you receive any professional treatment for these illnesses or injuries?
		Q0610: Do you think that you needed treatment?
17	Tunisia world health survey (WHS) (2003)	Q7004: The last time you [your child] needed health care, did you get health care?
		A4045: During the last 12 months, have you had any problems with your mouth and/or teeth (this includes problems with swallowing)?
18	World values survey (WVS) wave 6 (2010–2014)	V190: In the last 12 month, how often have you or your family gone without medicine or medical treatment that you needed?
	World values survey (WVS) wave 7 (2017–2021)	Q53: In the last 12 months, how often have your or your family…gone without medicine or medical treatment that you needed?

For example, the WHO Study on global AGEing and adult health (SAGE) asked a single question about unmet need, “The last time you needed health care, did you get health care?”, with an open time frame within the last 3 years, followed by two questions—one asking about the symptoms, illness, condition or event that was the basis for needing care, and one about the reasons for not getting that care (with 10 response categories). The Cambodia Elderly Study (CES) used four questions (see Table 2) before a question asking about the reason the respondent did not get health care (with 7 response categories). The Integrated Household Survey (IHS) in The Gambia also used two similar questions (“During the last 2 weeks, what symptoms has [NAME] suffered from…”? and “Did [NAME] consult a health provider for this illness/injury last 2 weeks for MAIN illness?”, before asking about the reasons for not getting care (7 response categories). The Commonwealth Fund International Health Policy Survey’s (CMWF) approach used four questions about unmet need related to prescription medications, medical visits, skipped tests or dentist visits—but all related to the barrier of cost (see Table 2).

The Thai Health and Welfare Study (HWS) asked questions about unmet need from outpatient or inpatient services: “UN1 (out-patient): Was there any time during the last 12 months when you were sick and needed a medical treatment but you did not receive it?” and “UN3 (in-patient): Was there any time during the last 12 months when you needed or were recommended by a medical doctor to admit to a health facility but you did not receive it?”.

The time period covered by questions also differed: some questions did not specify a time period (SAGE), while others did [for example, in the last 24 h (New Zealand Health Survey (NZHS)), last 2 weeks (ELSI-Brazil), last 30 days (SABE Colombia), last 12 months (Cambodia, SABE Ecuador, Myanmar 2016), or last 5 years (MHAS)]. This again adds variability into the resulting estimates. The question in the NZHS was even more precise—where the 24 h timeframe was further quantified as an event “in the past 12 months”.

The year of data collection for the studies ranged from 2001 to 2019. Some studies implemented only one cross-sectional wave (ELSI-Brazil, Myanmar Aging Study (MAS) and Mongolia SAGE), while some had sequential cross-sectional waves (new respondents at each wave: IHS, HWS and NZHS), while other studies had longitudinal waves of data collection (some respondents followed over time, for example, SAGE, PREHCO and MHAS).

The target study populations also varied by study—with the focus of this analysis on those aged 60 years and older. Some studies had no lower or upper age limit (HWS), while others had quite different age limits 15+ years (Tunisia Health Examination Survey), 16+ years (World Values Survey), or 65+ years (Commonwealth Fund International Health Policy Survey). A number of ageing studies were included with 50, 60 or 65 years as their lower age threshold.

These examples point to differences in the target universe of individuals used to derive the estimated prevalence of unmet health needs, which potentially reduces the comparability across studies. The question or set of questions, and wording of those questions along with response categories, likely add a degree of variability within and across countries in the final estimates—but overall point to levels of unmet need and challenges countries will face in equitably achieving UHC. Details about these questions and methods of generating the estimates are provided in Table 2, with additional detailed information in a Technical Note available through an author’s institute (https://extranet.who.int/kobe_centre/en/project-details/unmet_needs).

In an effort to provide comparable estimates within and across studies and world regions, these analyses focused on generating prevalence estimates for adults aged 60+ years. A set of unweighted and weighted estimates of unmet need were then generated for each question set by study, and according to age group, sex, and urban or rural residence. Due to uncertainties about study weights, and sub-population analyses (older adults), we present here the unweighted prevalence estimates of unmet health care need in populations aged 60+ years. Statistical significance for sex and location differences was set at *p* < 0.05.

## Results

**Fig. 2:**
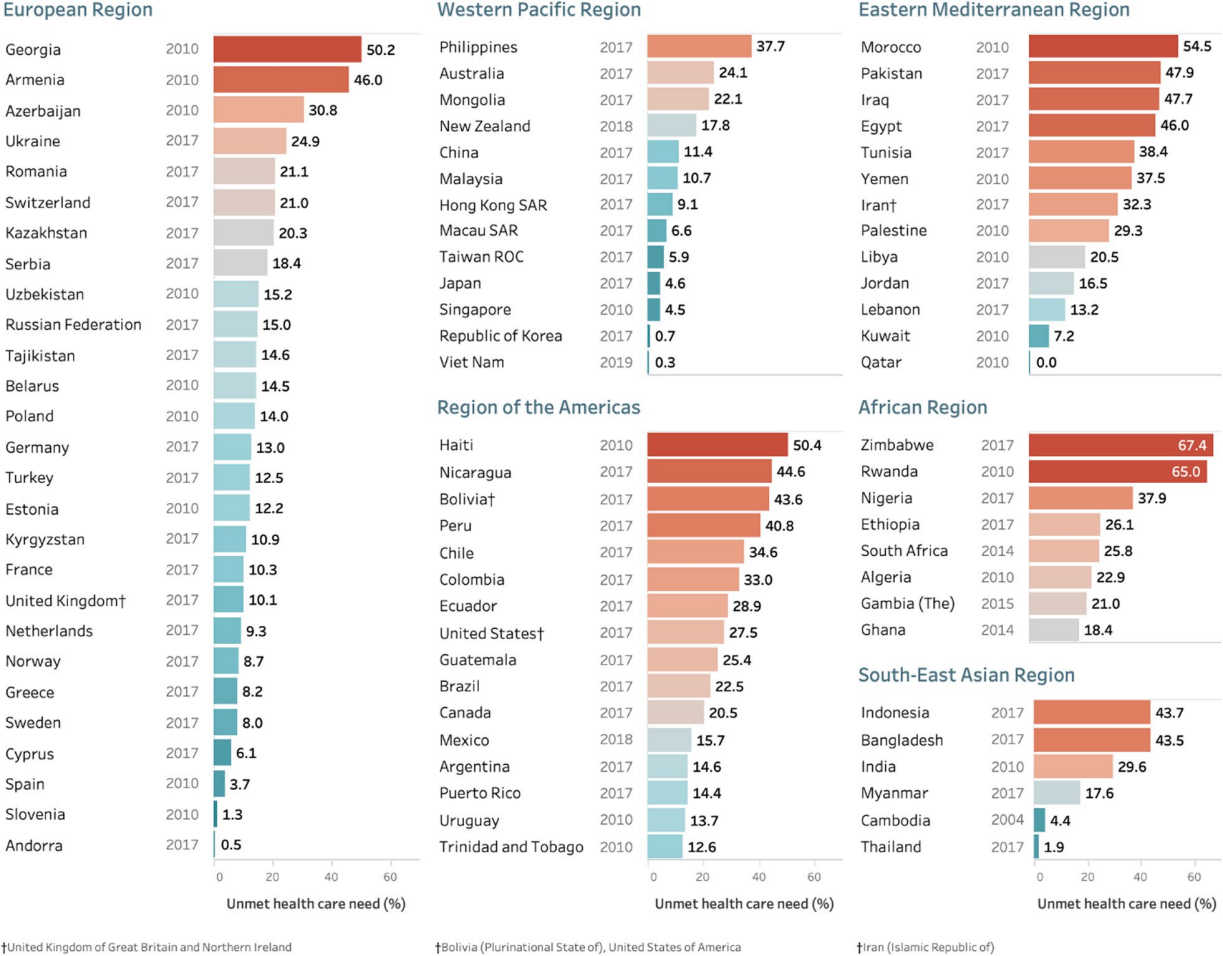
Prevalence of unmet health care need overall for adults aged 60+ years using the most recent study^‡^, by country and WHO region. ^‡^Where multiple studies are available in a country, data from the most recent year was used. *Note*: results are unweighted due to differences in weights provided for each study

Eighteen studies covering 83 low-, middle- and high-income countries were accessed, including *n* = 79,118 adults aged 60+ years with health care need. Using the most recent data for each country, the results point to low prevalence (less than 2%) of unmet need for the populations aged 60+ years in Andorra, Qatar, Republic of Korea, Slovenia, Thailand and Viet Nam. Twenty-two countries had prevalence estimates of 30% or higher (see Fig. 2). The prevalence of unmet health care need exceeded 50% in four countries: Georgia, Haiti, Morocco, Rwanda, and Zimbabwe.

**Fig. 3:**
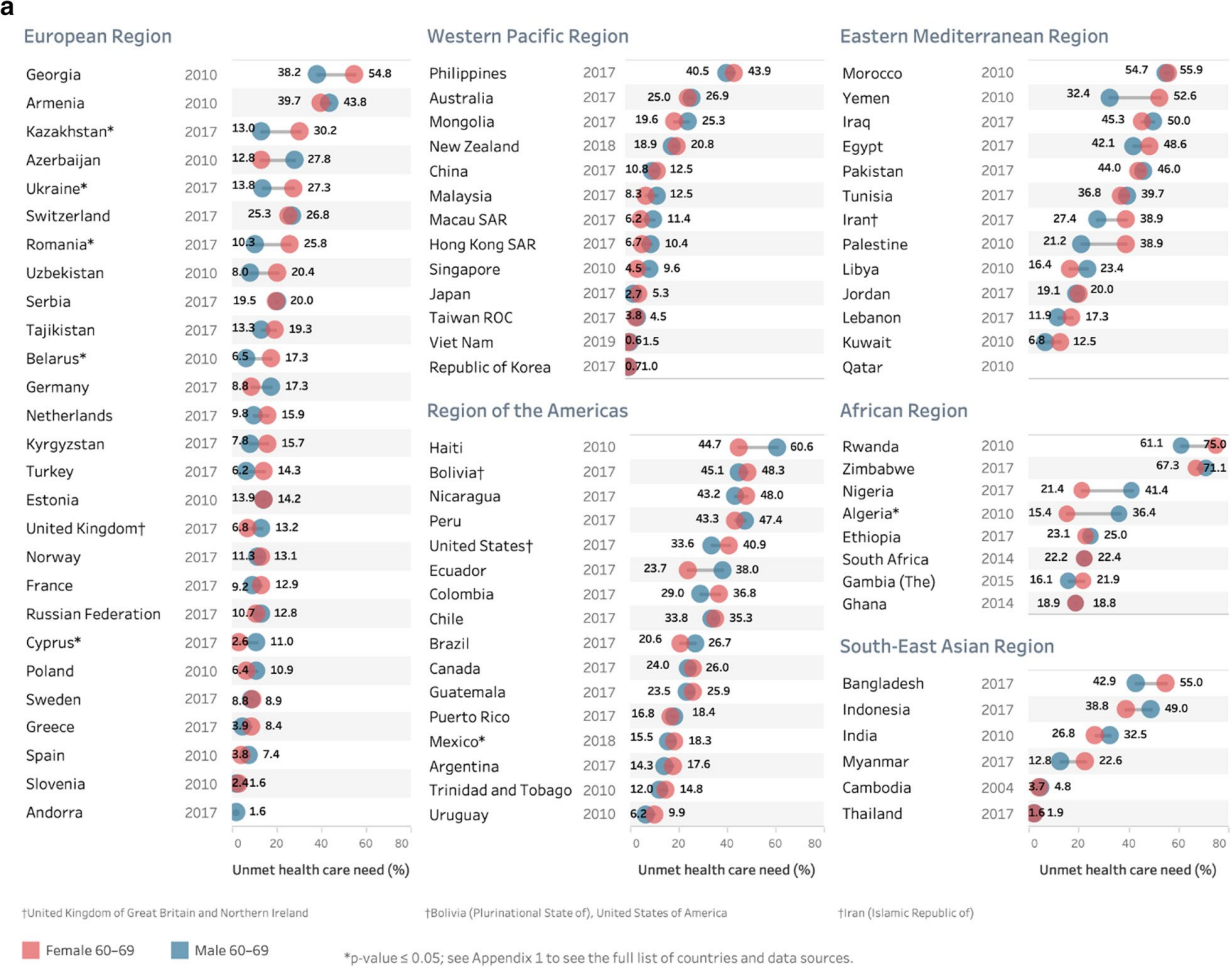

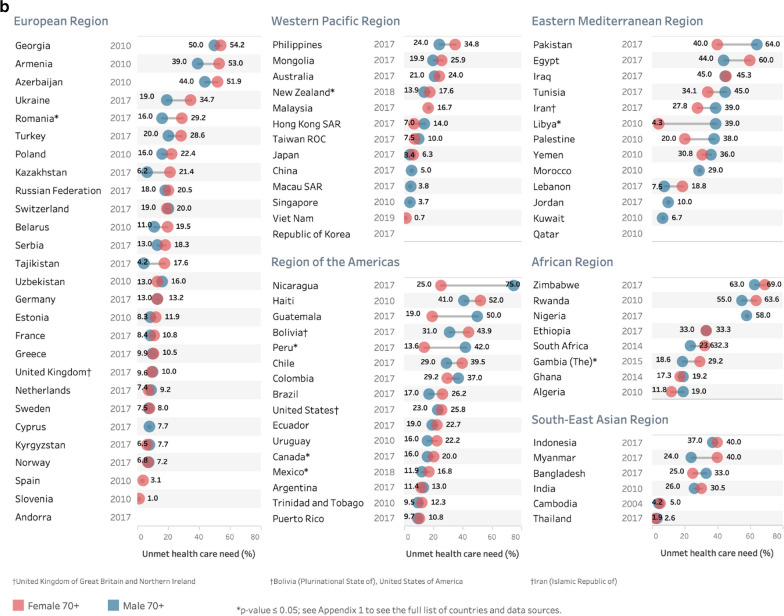
Prevalence of unmet health care need and for males and females aged **a **60–69 years and **b** 70+ years using the most recent study^‡^, by country and WHO region. ^‡^Where multiple studies/waves were available in a country, data from the most recent year was used. *Note:* results are unweighted

**Fig. 4:**
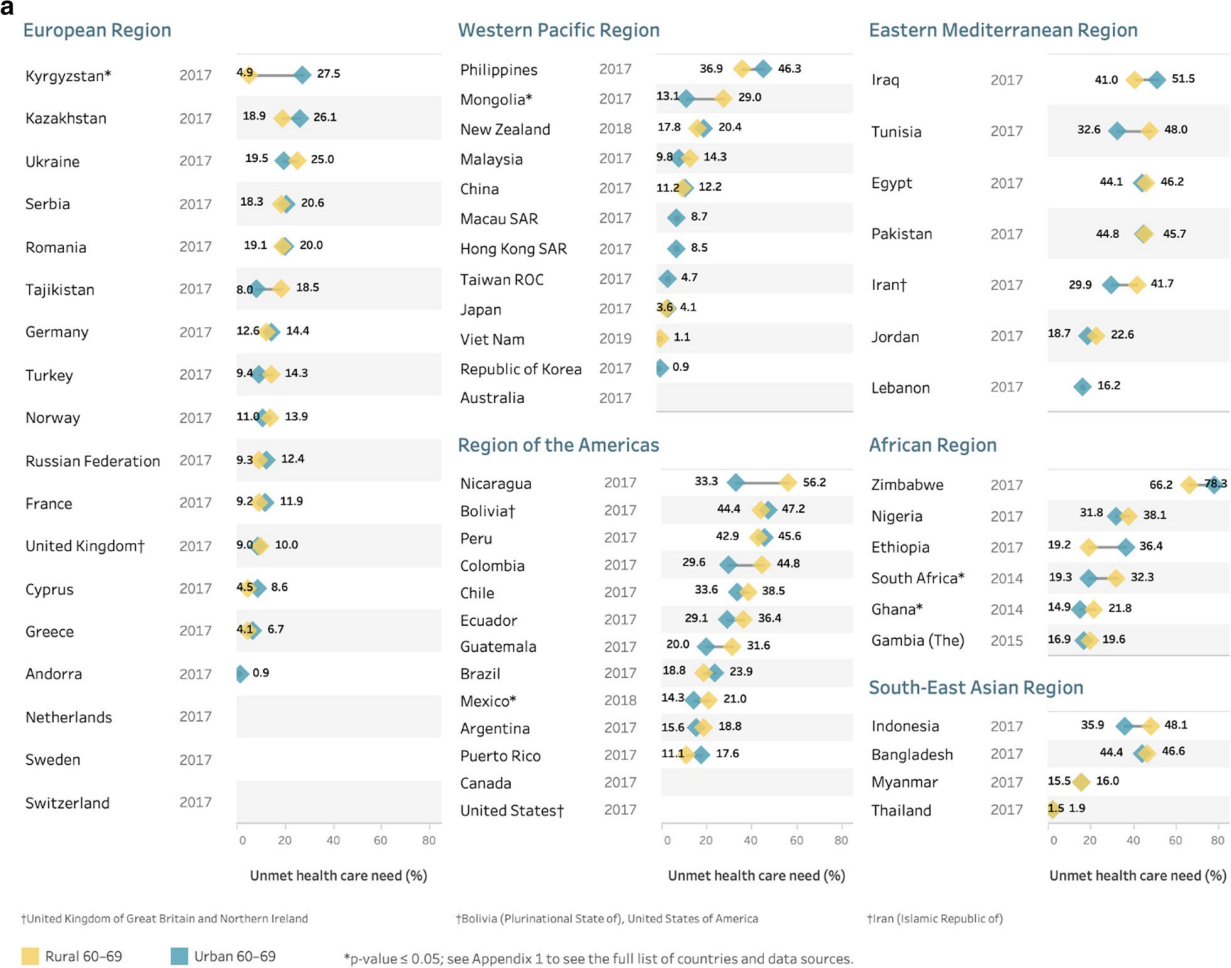

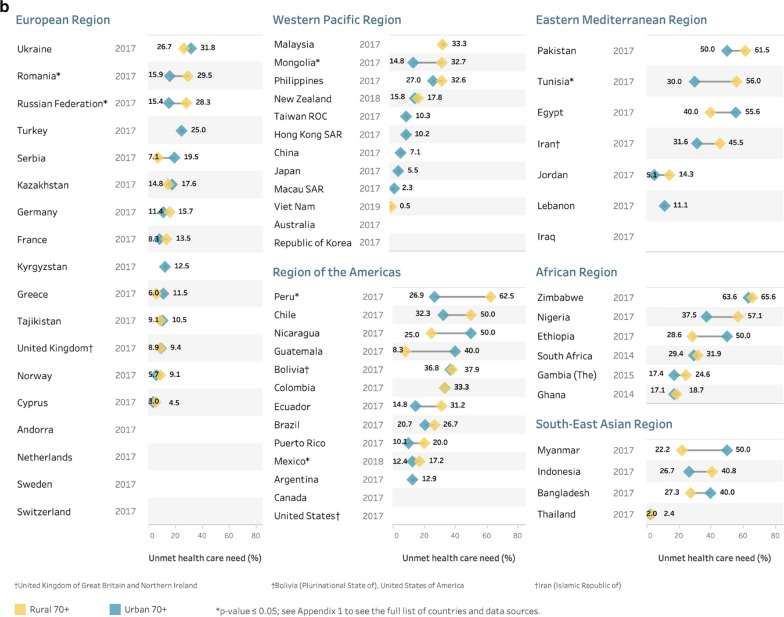
Prevalence of unmet health care need for urban or rural populations aged **a** 60–69 years and **b** 70 + years using the most recent study^‡^, by country and WHO region. ^‡^Where multiple studies were available in a country, data from the most recent year was used. *Note* results are unweighted. No plotted data means location information not available

Results presented in Figs. 2, 3 and 4 below were grouped by WHO regions (see more information at: www.who.int/about/who-we-are/regional-offices). Figure 2 includes the population aged 60+ years by country and region. The set of countries show gradients in unmet need within each region with the highest country prevalence above 50% in each region except the Western Pacific Region and South-East Asian Region (with the Philippines at 37.7% and Indonesia at 43.7%). A wide range in estimates are observed in countries in the African (18.4–67.4%), Eastern Mediterranean (0–54.5%) and European (0.5–50.2%) regions.

Results in Fig. 3a, b focus on two age groups (60–69 and 70+ years) for men and women, again with countries organized by WHO region. These figures show that the differences between women and men were generally small. Where differences were statistically significant (*)—in the 60–69 year group—five countries had higher rates of unmet need in women than men (Belarus, Kazakhstan, Mexico, Romania and Ukraine), and men in two countries, Algeria and Cyprus, had higher rates than women. In the 70+ age group, five countries had higher rates in women than men (Canada, Gambia, Mexico, New Zealand, and Romania) and men from two countries (Libya and Peru) had higher rates than women. A number of countries had large differences in prevalence rates between men and women—but did not reach statistical significance (likely due to low sample size in these age groups—see Additional file 1: Appendix table).

A number of studies did not include variables that allowed for examination of differences by location of residence, such as urban or rural locations. Where these data were available, many countries had small location-related differences while some had quite large differences (see Fig. 4a, b). Where statistically significant differences did exist in the 60–69 year old population (Fig. 4a), one country had higher unmet need in urban areas (Kyrgyzstan), while four countries had higher unmet need in rural areas (Ghana, Mexico, Mongolia, South Africa). For the 70+ population (Fig. 4b), higher unmet need in rural areas was seen in Mexico, Mongolia, Peru, Romania, Russia, and Tunisia.

In both age groups, the studies may have been underpowered to show statistical significance in the prevalence differences by sex and residence (see Additional file 1: Appendix 2). Thus, the prevalence may be worth investigating further in larger study samples.

**Fig. 5:**
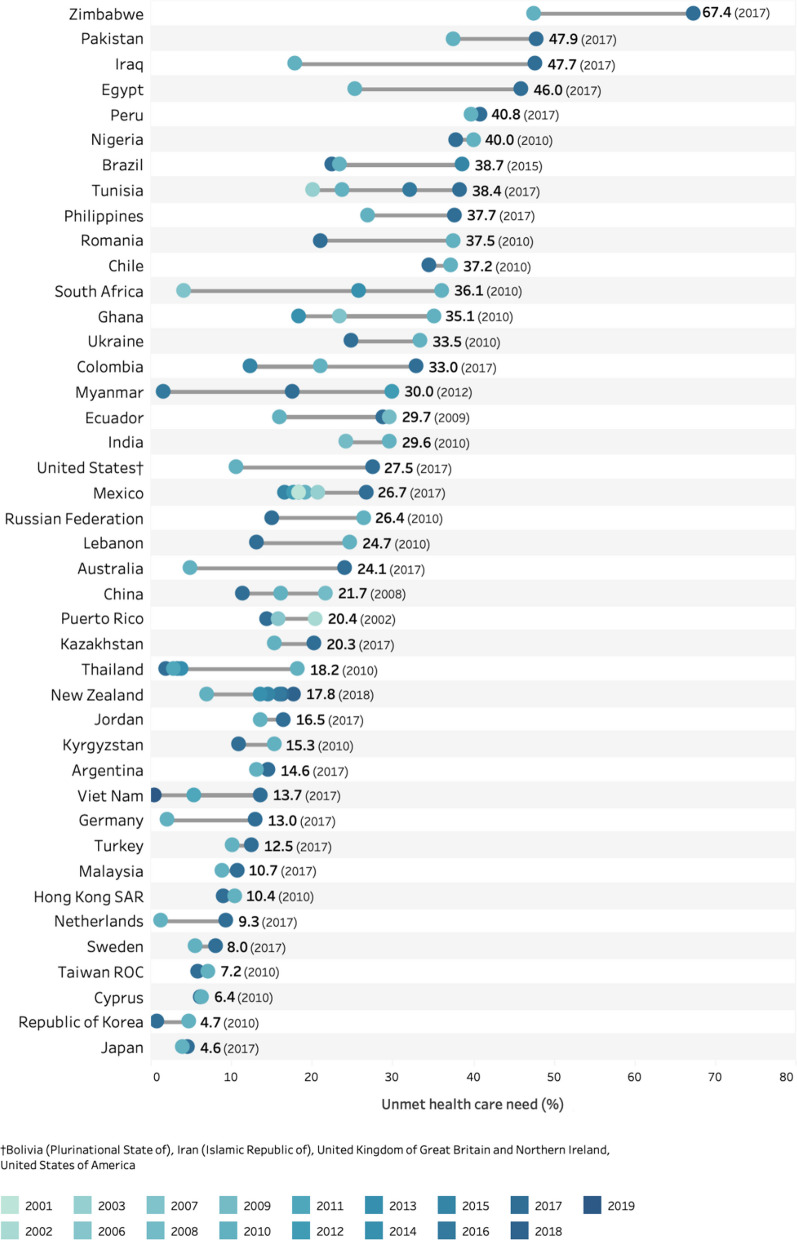
Overall prevalence of unmet need in the population aged 60+ years for different years of available data, by country^‡^. ^‡^*Note* only countries with more than one data point were included. Year of highest rate labelled. Results are unweighted

A number of studies in selected countries provided multiple years of data. Graded-colour data points are plotted for countries with data for multiple years (darker for more recent years) in Fig. 5, with a data label for the highest prevalence and year for each country. Countries like Australia, Egypt, Germany, Iraq, Netherlands, New Zealand, Pakistan, Philippines, Tunisia, United States, and Zimbabwe had higher prevalence in more recent years of available data (Fig. 5). Countries like China, Lebanon, Puerto Rico, Republic of Korea, Romania, Russia, and Ukraine may have lower prevalence in more recent years. Mixed patterns are seen in Ghana, Mexico, and Thailand—where a linear pattern of increasing or decreasing did not emerge and would require further investigation into the different tools and methods used to assess unmet need. An uncertain pattern emerges when comparing estimates from different studies within one country that might use different questions or study populations (for example in Brazil and Viet Nam).

**Fig. 6:**
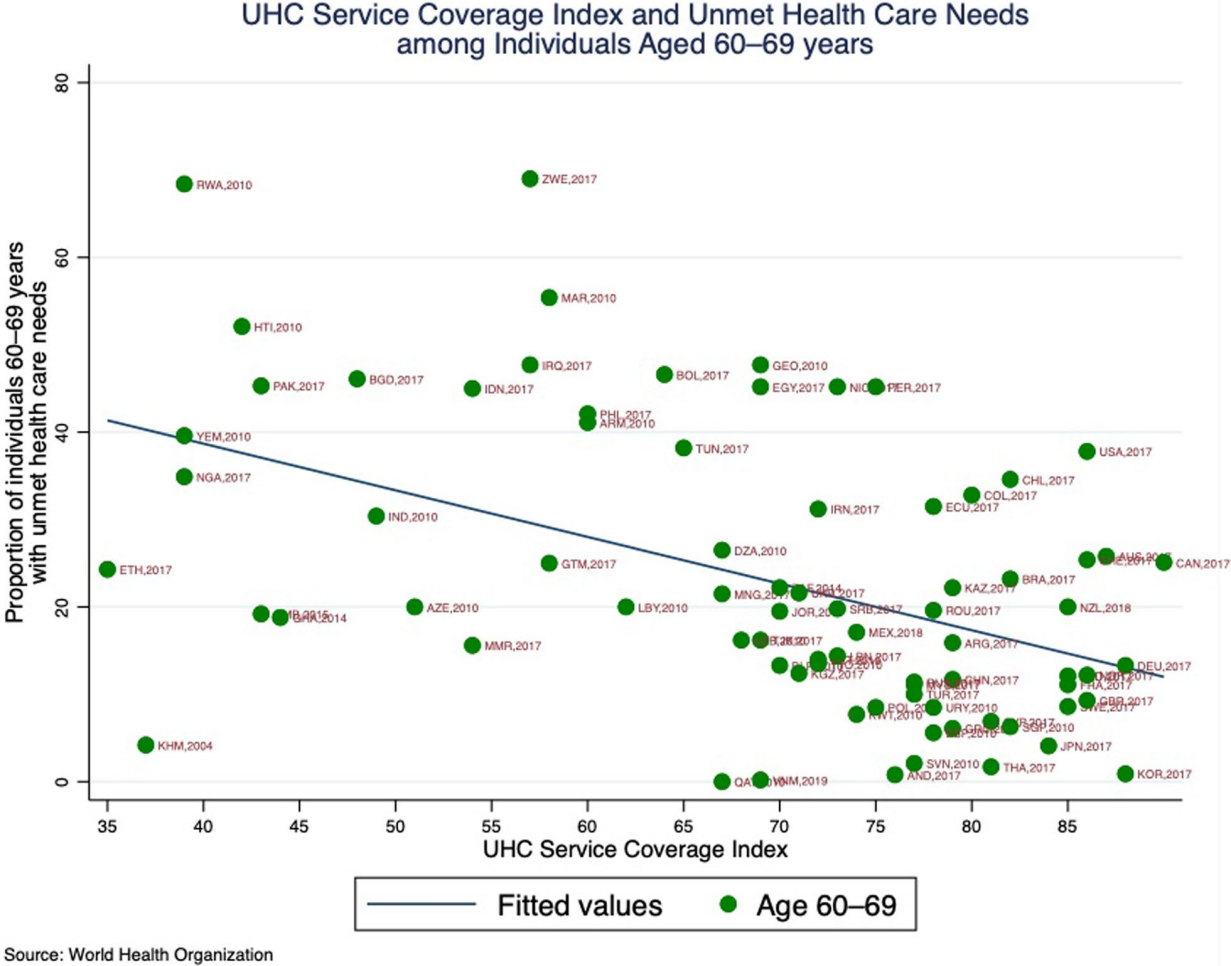
Point prevalence of self-reported unmet need for healthcare among adults aged 60–69 years, by UHC Service Coverage Index* at the year of the survey. *WHO UHC Service Coverage Index https://www.who.int/data/gho/data/indicators/indicator-details/GHO/uhc-index-of-service-coverage

To ensure an equitable path to UHC that includes older adults, a number of issues, including unmet health care need require further investigation and policy action. Leaving aside the complexity of determinants for unmet healthcare needs, currently available data can be used to examine the relationship between unmet need and UHC for policy and planning purposes. Progress toward UHC is being tracked using indices that capture both service coverage and financial protection [15] For example, WHO’s UHC Service Coverage Index incorporates 14 tracer indicators (covering reproductive, maternal, newborn and child health, infectious diseases, non-communicable diseases and service capacity and access) of service coverage into a single summary measure (www.who.int/data/gho/data/indicators/indicator-details/GHO/uhc-index-of-service-coverage). A higher index score suggests a better position for progress towards UHC. Looking at the relationship between unmet need in adults aged 60–69 years and this UHC index, the overall pattern is that levels of unmet health need tend to be lower in countries with higher values of the UHC Service Coverage Index ((r = – 0.47, *p*-value < 0.0001), at the global and regional levels (Fig. 6). This supports expectations that advancing UHC would lead to reductions in unmet health needs, and conversely, that addressing unmet health needs would improve levels of UHC service coverage.

For older adults, continued investment in both health and social care systems strengthening will be required [16] to address both unmet health and social care needs and ensure no one is left behind. However, the relative efficiency with which countries can translate their health spending into improved service coverage and financial protection varies considerably across the region, even in countries at similar levels of development.

## Discussion

A key objective for health policy and health systems, especially in countries working towards universal health coverage, is to provide access to good quality services and meet population health care needs. Assessing whether health systems succeed is made difficult by not only the variation on how need is defined and measured, but also on the lack of data whether the needs were met or unmet. For certain population groups, including older adult populations, a mismatch may exist between use of health care services and what they need. This leads to policy concern about systematic under-utilization of healthcare resources relative to their level of need, particularly when the consequences are poorer health outcomes from preventable causes.

Our study indicates that older adults residing in low-, middle- and high-income countries experienced delayed and foregone care. This ranged from no unmet need to a high prevalence of 67%. These rates are consistent with results from Kenya from 2016 in the population aged 16+ years (19.6%, [17]) and 14.5% in a study in the Korean population aged 19 years and older [18]. Twenty-six percent of the European Union population aged 15 years and older reported having an unmet need for health care in 2014 [19]. A Korean study of those aged 65+ years reported a prevalence of 17.4% [20]. Older adults with high care needs or multiple co-morbid conditions may have higher levels of adverse consequences from not being able to access care [21]. These rates may well increase in many countries as a result of the ongoing COVID-19 pandemic, with emerging evidence about unmet health care needs [22–24].

This study did not attempt direct comparisons across studies in these analyses and did not age standardize results. The nature of the questions and methods employed across studies would make cross-country comparisons challenging—and instead the focus was on generating reliable estimates for the prevalence of unmet need for older adults (60+) for each country from the available data.

We found variation in prevalence rates in the two age groups explored in this study—but no clear patterns emerged. Separate analyses (results not included here) found that countries in the European region generally had populations aged 70+ with higher or similar unmet need than younger age groups, whereas the age-related patterns were more mixed in countries in the other regions. For example, in the Americas only Chile and Guatemala had the highest unmet need in the population aged 70+. The differences by age group were small in all countries in the Western Pacific. In the 7th European Social Survey, including populations aged 25–75 years of age, younger age groups reported more unmet need [25].

Only a handful of countries had significant sex differences in those aged 60–69 and 70+ (for example, Romania and Mexico). A wider number of countries had sex differences in one age group, but not the other (Algeria, Belarus, Canada, Cyprus, Libya, Peru, Kazakhstan and Ukraine). Where non-significant differences were seen between the sexes—some countries had higher levels in men than women (Azerbaijan, Haiti, Indonesia) while others had higher levels in women than men (Egypt, Rwanda) and with some switching by age group (men in 60–69 and women in 70+ in Armenia; women in 60–69, men in 70+ in Nicaragua). Yet we do not know how meaningful these differences are—when a smaller number of countries had statistically significant sex differences in prevalence estimates.

A number of countries had significant differences in prevalence by urban or rural location of residence. Similar to sex differences, a small number of countries (9) had differences that reached statistical significance. An OECD summary of results from 31 countries showed that within a society, those with lower incomes experienced more barriers to accessing care than the richer counterparts—with some variations across countries and by the set of reasons provided for unmet needs [26]. These findings of a socio-economic gradient in unmet need for health care have been consistent over time [19, 25]. Estimates for unmet need may help to show the distribution of financial hardship across different sectors of the population in a variety of countries. In those countries with substantial differences by urban or rural location, that information provides some initial evidence to policy makers in their efforts to better target policies and programmes to ensure equal implementation of universal health coverage across different regions within countries.

These results are unique in providing estimates of unmet need for older adults in a wide range of countries from different world regions. Additional analyses for some of the studies included in this study could be undertaken to look at types of unmet need (for example, medical, dental, medication) and to examine barriers to care (cost, transport). Accessing additional longitudinal ageing and health study data, and undertaking analysis of longitudinal data, would be needed to investigate changes over time and causative factors.

### Comparability of estimates across countries

A number of differences in methodologies may influence the study-specific estimates and overall results. Studies included in this paper targeted different populations, ranging from no age limits in one study to 60+ years for a number of the studies. Where possible, we excluded those who reported no health needs—and the analyses used carefully determined denominators to generate robust estimates for each study.

The question wording differed considerably by survey—where the estimates generated here were based on examination of the full sets of questions in each study. Question ordering and phrasing may also contribute to differences in final prevalence estimates [27]. The questions that asked about need/unmet need also included a variety of timeframes—from care within 24 h in the last year, to any care in the last 3 years preceding the interview. The questions also ranged from health care “in general”—without specifying the type of care—to questions that included a more specific example (or examples) of types of care (medical, dental, surgical, mental health, medications). A number of studies included foregone care due to cost within the main question—meaning the question asked about not receiving a type of care because of cost (compared to other reasons or barriers like access, travel time, or wait time).

While this study provides prevalence estimates for a wide range of countries from recent studies, the challenge of fully quantifying levels of unmet need remains, particularly when there is no consensus on a definition—and sets of questions about unmet need have inconsistently used and applied in health and/or consumption surveys in low-and-middle-income countries. Some studies, in OECD, Commonwealth and European countries, have attempted to use the same questions in multiple countries—but even the wording of these questions differ when comparing the different studies.

### Limitations

While asking questions as part of health surveys is efficient, self-reported measures of unmet need for health care present a number of methodological challenges. A comprehensive approach to the issue would require: (1) an understanding of what people understand when answering a direct question about unmet need; (2) an understanding of the nature of a person’s specific needs based on her/his health status, illness and preferences; and/or (3) an assessment of whether these needs have been adequately met according to clinical standards factoring in options effectively available to patients. To achieve one of the pillars of universal health coverage, this would also include no financial hardship as a result of paying for needed services [28–30]. The next step would be to standardize the approach, so that surveys could capture this information using similar methods: either through *ex post facto* secondary data harmonization processes or *ex-ante* agreed standards for primary data collection instruments.

Unmet health care needs in these analyses include individuals who perceived a need for healthcare but did not seek or receive treatment; those who did not perceive a need for healthcare were not included. We attempted to assess “need” and met or unmet need where data was available. However, based on the available data and how unmet need was assessed in each study, the prevalence of unmet health care needs presented here may be underestimated to some extent. Moreover, the small sample size of older persons in some countries or studies (see Additional file 1: Appendix 2) may reduce the statistical power and increase the margin of error, resulting in non-significant differences in sex and area of residence. We also could not adjust for the moral hazard related to insurance coverage and perceptions about need or unmet need, which might overestimate the likelihood of unmet health care needs [31]. Finally, the validity of the estimates are influenced by the self-reported nature of the data from these health surveys. We do know what respondents have in mind when responding to these questions. Generating estimates of unmet health care need would be challenging for single diseases, and self-report does not easily lend itself to establishing a measure of (lack of) access to services for those with multi-morbidity or at a health system level for an entire population. More work is needed, but the relationship found between the unmet need estimates and UHC Service Coverage Index points to the possible validity of this measure as a rough indicator of (lack of) service access. These results can be used to begin policy dialogue about how to measure concrete progress on moving towards UHC and policy levers required to ensure does not leave any populations behind. Further to this, unmet social care need was not addressed in the results presented here, but could contribute as much or more to access issues for older people [10, 32].

The estimates presented in this report were not weighted. Applying the raw weights (as provided in the datasets) to estimate the prevalence of unmet health care need for a specific sub-population of interest may result in incorrect estimates. The raw weights are usually derived using the study sample and overall population size, often covering respondents from a wider range of age groups, rather than on the actual sample size of those aged 60+ years [33]. However, we compared unweighted prevalence with weighted prevalence using sample weights included in the datasets (see Additional file 1: Appendix 2) and saw no significant differences.

## Summary

These results are unique in providing estimates of unmet need for older adults in a wide range of countries from different world regions. It also highlights the challenges with standardizing measurement and provides important groundwork for working towards an agreed definition of unmet health care need. These factors will also help to generate evidence to inform policies aimed at supporting progress towards universal health coverage by 2030 (SDG target 3.8). Current metrics used to inform policy, UHC indices, may not adequately account for unmet health need—risking universality in health coverage especially for older and vulnerable populations Additional analyses for some of the studies included here could be undertaken to look at types of unmet need (for example, medical, dental, medication) and to examine barriers to care (cost, transport) to better target policy deliberations. Accessing additional longitudinal ageing and health study data, and undertaking analysis of longitudinal data, would be welcome to investigate changes over time and causative factors to better inform policy and programming.

### Supplementary Information

**Additional file 1**. Supplementary tables and analysis details.

## Data Availability

The data that support the findings of this study are available from each study on application/request. We applied for access to each dataset. Data are however available from the authors upon reasonable request and with permission of the respective study. Additional information will be available through https://extranet.who.int/kobe_centre/en/project-details/unmet_needs.

## Abbreviations

ELSI-Brazil: Brazilian Longitudinal Study of Aging
CES: Cambodia Elderly Survey
CMWF: Commonwealth Fund International Health Policy Survey
HWS: Thai Health and Welfare Study
IHS: Integrated Household Survey
MHAS: Mexico Health and Aging Study
MAS: Myanmar Aging Study
NZHS: New Zealand Health Survey
OECD: Organization for Economic Cooperation and Development
OP: Older Persons
PREHCO: Puerto Rican Elderly: Health Conditions Project
SABE: Survey on Health, Well-Being, and Aging
SAGE: Study of global AGEing and adult health
SHI: Social Health Insurance
THES: Tunisian Health Examination Survey
VNAS: Vietnam Ageing Survey
WHS: World Health Survey
WVS: World Values Survey
