# Supplementary material for: Prevalence of unmet health care need in older adults in 83 countries: measuring progressing towards universal health coverage in the context of global population ageing
Source: Popul Health Metr. 2023 Sep 15;21:15. doi: 10.1186/s12963-023-00308-8 (PMC10503154; doi:10.1186/s12963-023-00308-8)
Supplement: Supplementary file 1 — Additional file 1. Supplementary tables and analysis details. [file 12963_2023_308_MOESM1_ESM.docx]

Supplementary

Appendices

Appendix 1. List of countries and total samples

| **Countries** | | **Region** | **Survey** | **Year** | **N** | **N ≥ 60 years** |
| --- | --- | --- | --- | --- | --- | --- |
| 1 | Algeria | African Region | WVS* | 2010/14 | 1200 | 131 |
| 2 | Andorra | European Region | WVS | 2017/21 | 1004 | 222 |
| 3 | Argentina | Region of the Americas | WVS | 2010/14 | 1030 | 222 |
|  |  |  | WVS | 2017/21 | 1003 | 226 |
| 4 | Armenia | European Region | WVS | 2010/14 | 1100 | 272 |
| 5 | Australia | Western Pacific Region | CMWF | 2017 | 2500 | 600 |
|  |  |  | WVS | 2010/14 | 1477 | 590 |
|  |  |  | WVS | 2017/21 | 1813 | 765 |
| 6 | Azerbaijan | European Region | WVS | 2010/14 | 1002 | 120 |
| 7 | Bangladesh | South-East Asian Region | WVS | 2017/21 | 1200 | 92 |
| 8 | Belarus | European Region | WVS | 2010/14 | 1535 | 337 |
| 9 | Bolivia (Plurinational State of) | Region of the Americas | WVS | 2017/21 | 2067 | 264 |
| 10 | Brazil | Region of the Americas | ELSI | 2015 | 2067 | 1715 |
|  |  |  | WVS | 2010/14 | 1486 | 265 |
|  |  |  | WVS | 2017/21 | 1762 | 378 |
| 11 | Cambodia | South-East Asian Region | SEC | 2004 | 1762 | 30 |
| 12 | Canada | Region of the Americas | CMWF | 2017 | 4549 | 933 |
| 13 | Chile | Region of the Americas | WVS | 2010/14 | 1000 | 199 |
|  |  | Region of the Americas | WVS | 2017/21 | 1000 | 205 |
| 14 | **China** | Western Pacific Region | SAGE-W1 | 2008/10 | 15050 | 1288 |
|  |  |  | WVS | 2010/14 | 2300 | 370 |
|  |  |  | WVS | 2017/21 | 3036 | 607 |
| 15 | Colombia | Region of the Americas | WVS | 2010/14 | 1512 | 218 |
|  |  |  | WVS | 2017/21 | 1520 | 191 |
|  |  |  | SABE | 2015 | 3036 | 1,007 |
| 16 | Cyprus | European Region | WVS | 2010/14 | 1000 | 203 |
|  |  |  | WVS | 2017/21 | 1000 | 214 |
| 17 | Ecuador | Region of the Americas | WVS | 2010/14 | 1202 | 187 |
|  |  |  | WVS | 2017/21 | 1200 | 173 |
|  |  |  | SABE | 2009 | 5212 | 1,285 |
| 18 | Egypt | Eastern Mediterranean Region | WVS | 2010/14 | 1523 | 224 |
|  |  |  | WVS | 2017/21 | 1200 | 87 |
| 19 | Estonia | European Region | WVS | 2010/14 | 1533 | 483 |
| 20 | Ethiopia | African Region | WVS | 2017/21 | 1230 | 46 |
| 21 | France | European Region | CMWF | 2017 | 750 | 77 |
| 22 | Gambia (The) | African Region | IHS | 2015 | 52955 | 153 |
| 23 | Georgia | European Region | WVS | 2010/14 | 1202 | 257 |
| 24 | Germany | European Region | CMWF | 2017 | 751 | 97 |
|  |  |  | WVS | 2010/14 | 2046 | 663 |
|  |  |  | WVS | 2017/21 | 1528 | 509 |
| 25 | Ghana | African Region | SAGE-W1 | 2007/08 | 1528 | 571 |
|  |  |  | SAGE-W2 | 2014/15 | 4735 | 279 |
|  |  |  | WVS | 2010/14 | 1552 | 74 |
| 26 | Greece | European Region | WVS | 2017/21 | 1200 | 404 |
| 27 | Guatemala | Region of the Americas | WVS | 2017/21 | 1203 | 71 |
| 28 | Haiti | Region of the Americas | WVS | 2010/14 | 1996 | 113 |
| 29 | Hong Kong SAR | Western Pacific Region | WVS | 2010/14 | 1000 | 206 |
|  |  |  | WVS | 2017/21 | 2075 | 513 |
| 30 | India | South-East Asian Region | SAGE-W1 | 2009 | 12198 | 811 |
|  |  |  | WVS | 2010/14 | 4078 | 560 |
| 31 | Indonesia | South-East Asian Region | WVS | 2017/21 | 3200 | 316 |
| 32 | Iran (Islamic Republic of) | Eastern Mediterranean Region | WVS | 2017/21 | 1499 | 158 |
| 33 | Iraq | Eastern Mediterranean Region | WVS | 2010/14 | 1200 | 84 |
|  |  |  | WVS | 2017/21 | 1200 | 107 |
| 34 | Japan | Western Pacific Region | WVS | 2010/14 | 2443 | 854 |
|  |  |  | WVS | 2017/21 | 1353 | 581 |
| 35 | Jordan | Eastern Mediterranean Region | WVS | 2010/14 | 1200 | 154 |
|  |  |  | WVS | 2017/21 | 1203 | 200 |
| 36 | Kazakhstan | European Region | WVS | 2010/14 | 1500 | 188 |
|  |  |  | WVS | 2017/21 | 1276 | 143 |
| 37 | Kuwait | Eastern Mediterranean Region | WVS | 2010/14 | 1303 | 118 |
| 38 | Kyrgyzstan | European Region | WVS | 2010/14 | 1500 | 118 |
|  |  |  | WVS | 2017/21 | 1200 | 165 |
| 39 | Lebanon | Eastern Mediterranean Region | WVS | 2010/14 | 1200 | 93 |
|  |  |  | WVS | 2017/21 | 1200 | 167 |
| 40 | Libya | Eastern Mediterranean Region | WVS | 2010/14 | 2131 | 171 |
| 41 | Macau SAR | Western Pacific Region | WVS | 2017/21 | 1023 | 345 |
| 42 | Malaysia | Western Pacific Region | WVS | 2010/14 | 1300 | 123 |
|  |  |  | WVS | 2017/21 | 1313 | 84 |
| 43 | Mexico | Region of the Americas | SAGE-W1 | 2010 | 2742 | 304 |
|  |  |  | SAGE-W2 | 2014 | 4665 | 479 |
|  |  |  | WVS | 2010/14 | 2000 | 207 |
|  |  |  | WVS | 2017/21 | 1739 | 339 |
|  |  |  | MHAS | 2001 | 12235 | 1,192 |
|  |  |  | MHAS | 2003 | 11539 | 1,453 |
|  |  |  | MHAS | 2012 | 13608 | 1,614 |
|  |  |  | MHAS | 2015 | 13253 | 1,609 |
|  |  |  | MHAS | 2018 | 14805 | 1,423 |
| 44 | Mongolia | Western Pacific Region | MiniSAGE | 2017 | 983 | 194 |
| 45 | Morocco | Eastern Mediterranean Region | WVS | 2010/14 | 1200 | 121 |
| 46 | Myanmar | South-East Asian Region | MAS | 2012 | 4080 | 268 |
|  |  |  | Survey on Accessing Healthcare to Older Population in Myanmar | 2016 | 1000 | 10 |
|  |  |  | WVS | 2017/21 | 1200 | 131 |
| 47 | Netherlands | European Region | CMWF | 2017 | 750 | 70 |
|  |  | European Region | WVS | 2010/14 | 1902 | 772 |
| 48 | New Zealand | Western Pacific Region | CMWF | 2017 | 500 | 116 |
|  |  |  | WVS | 2010/14 | 841 | 282 |
|  |  |  | WVS | 2017/21 | 1057 | 533 |
|  |  |  | HS | 2014/15 | 13497 | 539 |
|  |  |  | HS | 2015/16 | 13781 | 632 |
|  |  |  | HS | 2016/17 | 13958 | 695 |
|  |  |  | HS | 2017/18 | 13869 | 721 |
|  |  |  | HS | 2018/19 | 13572 | 787 |
| 49 | Nicaragua | Region of the Americas | WVS | 2017/21 | 1200 | 74 |
| 50 | Nigeria | African Region | WVS | 2010/14 | 1759 | 65 |
|  |  |  | WVS | 2017/21 | 1237 | 58 |
| 51 | Norway | European Region | CMWF | 2017 | 750 | 65 |
| 52 | Pakistan | Eastern Mediterranean Region | WVS | 2010/14 | 1200 | 48 |
|  |  |  | WVS | 2017/21 | 1995 | 103 |
| 53 | Palestine | Eastern Mediterranean Region | WVS | 2010/14 | 1000 | 82 |
| 54 | Peru | Region of the Americas | WVS | 2010/14 | 1210 | 171 |
|  |  |  | WVS | 2017/21 | 1400 | 184 |
| 55 | Philippines | Western Pacific Region | WVS | 2010/14 | 1200 | 197 |
|  |  |  | WVS | 2017/21 | 1200 | 228 |
| 56 | Poland | European Region | WVS | 2010/14 | 966 | 272 |
| 57 | Puerto Rico | Region of the Americas | WVS | 2017/21 | 1127 | 406 |
|  |  |  | PREHCO 1 | 2002/03 | 966 | 872 |
|  |  |  | PREHCO 2 | 2006/07 | 966 | 499 |
| 58 | Qatar | Eastern Mediterranean Region | WVS | 2010/14 | 1060 | 70 |
| 59 | Republic of Korea | Western Pacific Region | WVS | 2010/14 | 1200 | 212 |
|  |  |  | WVS | 2017/21 | 1245 | 278 |
| 60 | Romania | European Region | WVS | 2010/14 | 1503 | 440 |
|  |  |  | WVS | 2017/21 | 1257 | 406 |
| 61 | Russian Federation | European Region | SAGE-W1 | 2010 | 4947 | 560 |
|  |  |  | WVS | 2010/14 | 2500 | 593 |
|  |  |  | WVS | 2017/21 | 1810 | 428 |
| 62 | Rwanda | African Region | WVS | 2010/14 | 1527 | 60 |
| 63 | Serbia | European Region | WVS | 2017/21 | 1046 | 275 |
| 64 | Singapore | Western Pacific Region | WVS | 2010/14 | 1972 | 368 |
| 65 | Slovenia | European Region | WVS | 2010/14 | 1069 | 310 |
| 66 | South Africa | African Region | SAGE-W1 | 2007/08 | 1069 | 71 |
|  |  |  | SAGE-W2 | 2014/15 | 3180 | 141 |
|  |  |  | WVS | 2010/14 | 3531 | 277 |
| 67 | Spain | European Region | WVS | 2010/14 | 1189 | 328 |
| 68 | Sweden | European Region | CMWF | 2017 | 7000 | 544 |
|  |  |  | WVS | 2010/14 | 1206 | 384 |
| 69 | Switzerland | European Region | CMWF | 2017 | 3238 | 668 |
| 70 | Taiwan ROC | Western Pacific Region | WVS | 2010/14 | 1238 | 258 |
|  |  |  | WVS | 2017/21 | 1223 | 357 |
| 71 | Tajikistan | European Region | WVS | 2017/21 | 1200 | 158 |
| 72 | Thailand | South-East Asian Region | WVS | 2010/14 | 1200 | 148 |
|  |  |  | WVS | 2017/21 | 1500 | 258 |
|  |  |  | HWS | 2011 | 56068 | 335 |
|  |  |  | HWS | 2013 | 56691 | 470 |
|  |  |  | HWS | 2015 | 112156 | 896 |
|  |  |  | HWS | 2017 | 53139 | 268 |
| 73 | Trinidad and Tobago | Region of the Americas | WVS | 2010/14 | 999 | 271 |
| 74 | Tunisia | Eastern Mediterranean Region | THES | 2016 | 10158 | 663 |
|  |  |  | WHS | 2003 | 5300 | 159 |
|  |  |  | WVS | 2010/14 | 1205 | 172 |
|  |  |  | WVS | 2017/21 | 1208 | 214 |
| 75 | Turkey | European Region | WVS | 2010/14 | 1605 | 159 |
|  |  |  | WVS | 2017/21 | 2415 | 73 |
| 76 | Ukraine | European Region | WVS | 2010/14 | 1500 | 412 |
|  | Ukraine | European Region | WVS | 2017/21 | 1289 | 353 |
| 77 | United Kingdom of Great Britain and Northern Ireland | European Region | CMWF | 2017 | 753 | 76 |
| 78 | United States of America | Region of the Americas | CMWF | 2017 | 1392 | 382 |
|  |  |  | WVS | 2010/14 | 2232 | 662 |
|  |  |  | WVS | 2017/21 | 2596 | 536 |
| 79 | Uruguay | Region of the Americas | WVS | 2010/14 | 1000 | 248 |
| 80 | Uzbekistan | European Region | WVS | 2010/14 | 1500 | 151 |
| 81 | Viet Nam | Western Pacific Region | WVS | 2017/21 | 1200 | 73 |
|  |  |  | VNAS | 2011 | 1500 | 1012 |
|  |  |  | OP&SHI | 2019 | 1500 | 758 |
| 82 | Yemen | Eastern Mediterranean Region | WVS | 2010/14 | 1000 | 80 |
| 83 | Zimbabwe | African Region | WVS | 2010/14 | 1500 | 101 |
|  |  |  | WVS | 2017/21 | 1215 | 176 |

Appendix 2a. Survey weighting

| **No** | **Study** | **Weighting** |
| --- | --- | --- |
| 1 | Brazilian Longitudinal Study of Aging (ELSI-Brazil) | weight=peso_calibrado_n, strata=estrato, cluster=UPA |
| 2 | Cambodia Elderly Survey (CES)^1^ | weight=newwt |
| 3 | Commonwealth Fund survey (CMWF) | weight=weight |
| 4 | Integrated Household survey (IHS) | No weighting |
| 5 | Mexico Health and Aging Study (MHAS) | Wave 1 2001: fac_per; Wave 2 2003: r2wtresp; Wave 3 2012: factori_12; Wave 4 2015: factori_15; Wave 5 2018: factori_18 |
| 6 | Myanmar Aging Study (MAS) | weight=weight_respondent |
| 7 | Myanmar Survey on Accessing Healthcare to Older Persons (2016) | No weighting |
| 8 | Mongolia Mini SAGE | No weighting |
| 9 | New Zealand Health Survey (NZHS) | Finalwgt_Final sample weight |
| 10 | Puerto Rican Elderly: Health Conditions (PREHCO) | Wave 1: weight=FAC_T, strata=ESTRATO, cluster=SECCION  Wave 2: weight=FACTORT, strata=ESTRATO, cluster=SECCION |
| 11 | Study of global AGEing and adult health (SAGE) | weight=pweight, strata=strata, cluster=q0101b |
| 12 | Survey on Health, Well-Being, and Aging (SABE) Ecuador | No weighting |
|  | Survey on Health, Well-Being, and Aging (SABE) Colombia | weight=ffinal_AJUSTAF, strata=ESTRATODISEÑO, cluster=upm |
| 13 | Sustainable Health Financing (SHIFT)(Timor-Leste) | No weighting |
| 14 | Thai Health and Welfare Survey (HWS) | weight=newwt |
| 15 | Tunisian Health Examination Survey (THES) | weight=weight_final, cluster=QICLUSTER |
| 16 | Vietnam Ageing Survey (VNAS) | PSUid |
| 17 | Survey on Older Persons and Social Health Insurance (OP&SHI) | village_id |
| 18 | Tunisia World Health Survey | weight=pweight, strata=strata, cluster=PSU |
| 19 | World Values Survey (WVS) | Wave 6: weight=V258  Wave 7: weight=W_WEIGHT |
| 1 Knodel, John E., Souvan Kiry Kim, Zachary S. Zimmer, and Sina Puch. 2005. "Older Persons in Cambodia: A Profile from the 2004 Survey of Elderly." PSC Research Report No. 05-576. 5 2005. | | |

Appendix 2b. Prevalence of unmet need by sex, age group and location (weighted and unweighted, where study weights available).

On request.

| **Table B1:** Unmet need by selected respondent characteristics, by country **(restrict to population aged 30+ years)** | | | | | | | |  |  |  |  |
| --- | --- | --- | --- | --- | --- | --- | --- | --- | --- | --- | --- |
| **Country** | **Survey** | **Year** | **N Unmet need (≥30 years)** | **Prevalence of unmet need (95% CI) (≥30 years)** | **PREVALENCE of Unmet need by sex, age group and location** | | | | | |  |
|  |  |  |  |  | **Sex** | |  | | **Residence** | |  |
|  |  |  |  |  | **Men** | **Women** | **60-69** | **70+** | **Urban** | **Rural** | **Notes** |
| Algeria | WVS | 2010/14 | 766 | 19.3 (16.4–22.1) | **22.8** | **15.2** | **26.5** | **16.7** | NA | NA | unweighted |
| **Algeria** | **WVS** | **2010/14** | **766** | **19.3 (16.4–22.1)** | **22.8** | **15.2** | **26.5** | **16.7** | **NA** | **NA** | **weighted** |
| Andorra | WVS | 2017/21 | 846 | 2.0 (1.1–3.0) | 1.4 | 2.6 | 0.8 | 0 | 2 | 2.2 | unweighted |
| **Andorra** | **WVS** | **2017/21** | **846** | **2.0 (1.1–3.0)** | **1.4** | **2.6** | **0.8** | **0** | **2** | **2.2** | **weighted** |
| Argentina | WVS | 2010/14 | 745 | 12.3 (9.9–14.7) | **11.1** | **13.3** | **12.2** | **14.3** | NA | NA | unweighted |
| **Argentina** | **WVS** | **2010/14** | **745** | **12.3 (9.7–14.9)** | **11.4** | **13** | **14.9** | **13.3** | **NA** | **NA** | **weighted** |
| Argentina | WVS | 2017/21 | 705 | 16.0 (13.2–18.7) | 16.1 | 15.8 | 15.9 | 12 | 16.3 | 12.3 | unweighted |
| **Argentina** | **WVS** | **2017/21** | **705** | **18.1 (14.7–21.6)** | **18.8** | **17.5** | **16.6** | **12.6** | **19.1** | **10.4** | **weighted** |
| Armenia | WVS | 2010/14 | 859 | 34.8 (31.6–38.0) | **31.3** | **36.5** | **41.4** | **49.1** | NA | NA | unweighted |
| **Armenia** | **WVS** | **2010/14** | **859** | **34.6 (31.2–38.0)** | **31.8** | **37.1** | **43.2** | **48.8** | **NA** | **NA** | **weighted** |
| Australia | CMWF | 2017 | 600 | 24.1 (22.4–25.8) | 23.6 | 24.4 | 25.8 | 22.8 | NA | NA | unweighted |
| **Australia** | **CMWF** | **2017** | **600** | **27.2 (23.6–30.7)** | **24.8** | **29.0** | **25.9** | **27.6** | **NA** | **NA** | **weighted** |
| Australia | WVS | 2010/14 | 1,325 | 7.3 (5.9–8.7) | **6.2** | **8.1** | **6.1** | **3.5** | NA | NA | unweighted |
| **Australia** | **WVS** | **2010/14** | **1,325** | **8.7 (6.7–10.6)** | **8.1** | **9.3** | **6.2** | **3.6** | **NA** | **NA** | **weighted** |
| Australia | WVS | 2017/21 | 1,634 | 8.7 (7.3–10.1) | 7.8 | 9.4 | 5.3 | 3.9 | 8.4 | 10.2 | unweighted |
| **Australia** | **WVS** | **2017/21** | **1,634** | **10.5 (8.5–12.5)** | **10.4** | **10.8** | **7.2** | **3.7** | **10.4** | **11.3** | **weighted** |
| Azerbaijan | WVS | 2010/14 | 720 | 27.4 (24.1–30.7) | **27.5** | **27.3** | **20** | **48.9** | NA | NA | unweighted |
| **Azerbaijan** | **WVS** | **2010/14** | **720** | **31.4 (27.4–35.3)** | **29.8** | **32.7** | **21.5** | **44.5** | **NA** | **NA** | **weighted** |
| Bangladesh | WVS | 2017/21 | 793 | 37.8 (34.4–41.1) | 34.2 | 42.5 | 46.1 | 31.2 | 37.4 | 37.8 | unweighted |
| **Bangladesh** | **WVS** | **2017/21** | **793** | **37.8 (34.4–41.1)** | **34.2** | **42.5** | **46.1** | **31.2** | **37.4** | **37.8** | **weighted** |
| Belarus | WVS | 2010/14 | 1,148 | 10.2 (8.4–11.9) | **8.2** | **11.6** | **13.3** | **16.5** | NA | NA | unweighted |
| **Belarus** | **WVS** | **2010/14** | **1,148** | **10.5 (8.7–12.4)** | **8.4** | **12.1** | **13.8** | **16.7** | **NA** | **NA** | **weighted** |
| Bolivia (Plurinational State of) | WVS | 2017/21 | 1,303 | 42.5 (39.8–45.2) | 37.2 | 47.8 | 46.6 | 37.2 | 42.3 | 43.6 | unweighted |
| **Bolivia (Plurinational State of)** | **WVS** | **2017/21** | **1,303** | **42.5 (39.8–45.2)** | **37.2** | **47.8** | **46.6** | **37.2** | **42.3** | **43.6** | **weighted** |
| Brazil | ELSI | 2015/16 | 2821 | 40.4 (39.3–41.6) | 37.2 | 42.3 | 39.2 | 38.2 | 39.9 | 43.6 | unweighted |
| **Brazil** | **ELSI** | **2015/16** | **2821** | **39.7 (37.2–42.2)** | **35.2** | **42.8** | **37.8** | **37.1** | **39.0** | **43.6** | **weighted** |
| Brazil | WVS | 2010/14 | 1,094 | 26.3 (23.7–28.9) | **20.6** | **29.8** | **27.1** | **16.8** | NA | NA | unweighted |
| **Brazil** | **WVS** | **2010/14** | **1,094** | **25.4 (22.8–28.0)** | **20.6** | **29.8** | **26** | **17.3** | **NA** | **NA** | **weighted** |
| Brazil | WVS | 2017/21 | 1,306 | 26.3 (23.9–28.7) | 24.6 | 27.7 | 23.2 | 21.3 | 26.2 | 26.6 | unweighted |
| **Brazil** | **WVS** | **2017/21** | **1,306** | **26.2 (23.8–28.7)** | **24.4** | **27.8** | **23.1** | **21.6** | **26.3** | **26.1** | **weighted** |
| **Cambodia** | **CES** | **2004** | **30** | **3.8 (2.5–5.8)** | **3.7** | **3.9** | **4.0** | **3.6** | **4.0** | **3.8** | **weighted** |
| Canada | CMWF | 2017 | 933 | 20.5 (19.4–21.7) | 18.5 | 21.7 | 25.1 | 18.7 | NA | NA | unweighted |
| **Canada** | **CMWF** | **2017** | **933** | **21.8 (20.1–23.4)** | **20.6** | **22.7** | **26.4** | **19.5** | **NA** | **NA** | **weighted** |
| Chile | WVS | 2010/14 | 764 | 25.7 (22.6–28.8) | **25.3** | **26** | **35.1** | **41.2** | NA | NA | unweighted |
| **Chile** | **WVS** | **2010/14** | **764** | **25.7 (22.6–28.8)** | **25.3** | **26** | **35.1** | **41.2** | **NA** | **NA** | **weighted** |
| Chile | WVS | 2017/21 | 821 | 25.5 (22.5–28.5) | 23.9 | 26.9 | 34.6 | 34.7 | 24.1 | 33.6 | unweighted |
| **Chile** | **WVS** | **2017/21** | **821** | **26.7 (22.7–30.7)** | **25.4** | **27.9** | **32.5** | **34.3** | **25.4** | **33.6** | **weighted** |
| China | SAGE-W1 | 2008/10 | 2164 | 19.4 (18.7-20.2) | 19.9 | 19.1 | 19.7 | 23.8 | 15.9 | 22.8 | unweighted |
| **China** | **SAGE-W1** | **2008/10** | **2164** | **16.1 (14.3-18.0)** | **16.1** | **16.1** | **18.8** | **23.6** | **13.4** | **18.2** | **weighted** |
| China | WVS | 2010/14 | 1,834 | 10.8 (9.2–12.3) | **11** | **10.6** | **14.7** | **20.9** | NA | NA | unweighted |
| **China** | **WVS** | **2010/14** | **1,834** | **10.6 (9.0–12.3)** | **10.6** | **10.6** | **13** | **20.2** | **NA** | **NA** | **weighted** |
| China | WVS | 2017/21 | 2,476 | 8.9 (7.8–10.0) | 8.5 | 9.3 | 11.7 | 3.6 | 9.1 | 8.6 | unweighted |
| **China** | **WVS** | **2017/21** | **2,476** | **8.8 (7.5–10.1)** | **8.2** | **9.4** | **11.6** | **1.7** | **9.2** | **8.3** | **weighted** |
| Colombia | SABE | 2015 | **1007** | **53.3(47.2 - 59.4)** | **48.5** | **56.7** | **55.2** | **51.4** | **53.2** | **54.0** | **weighted** |
| Colombia | WVS | 2010/14 | 1,038 | 29.8 (27.0–32.6) | **29.4** | **30.2** | **20.9** | **21.4** | NA | NA | unweighted |
| **Colombia** | **WVS** | **2010/14** | **1,038** | **29.8 (27.0–32.6)** | **29.4** | **30.2** | **20.9** | **21.4** | **NA** | **NA** | **weighted** |
| Colombia | WVS | 2017/21 | 961 | 36.5 (33.5–39.6) | 35.7 | 37.4 | 32.8 | 33.3 | 34.2 | 43.6 | unweighted |
| **Colombia** | **WVS** | **2017/21** | **961** | **36.5 (33.5–39.6)** | **35.7** | **37.4** | **32.8** | **33.3** | **34.2** | **43.6** | **weighted** |
| Cyprus | WVS | 2010/14 | 676 | 10.2 (7.9–12.5) | **6.5** | **13.6** | **6** | **6.9** | NA | NA | unweighted |
| **Cyprus** | **WVS** | **2010/14** | **676** | **9.4 (6.9–11.9)** | **6.2** | **12.5** | **6.4** | **7.5** | **NA** | **NA** | **weighted** |
| Cyprus | WVS | 2017/21 | 733 | 12.3 (9.9–14.7) | 13.6 | 11.2 | 6.9 | 3.6 | 14.7 | 8.8 | unweighted |
| **Cyprus** | **WVS** | **2017/21** | **733** | **8.3 (6.4–10.2)** | **9.5** | **7.3** | **5.2** | **3.7** | **9.9** | **5.4** | **weighted** |
| Ecuador | SABE | 2009 | 1,285 | 42.1 (40.4-43.9) | 39.5 | 44.0 | 44.0 | 40.5 | 38.0 | 47.6 | unweighted |
| Ecuador | WVS | 2010/14 | 794 | 15.0 (12.5–17.5) | **15.3** | **14.7** | **18.3** | **10.7** | NA | NA | unweighted |
| **Ecuador** | **WVS** | **2010/14** | **794** | **15.0 (12.5–17.5)** | **15.3** | **14.7** | **18.3** | **10.7** | **NA** | **NA** | **weighted** |
| Ecuador | WVS | 2017/21 | 805 | 30.5 (27.4–33.7) | 31.1 | 30.1 | 31.5 | 20.9 | 30.7 | 30.2 | unweighted |
| **Ecuador** | **WVS** | **2017/21** | **805** | **30.5 (27.4–33.7)** | **31.1** | **30.1** | **31.5** | **20.9** | **30.7** | **30.2** | **weighted** |
| Egypt | WVS | 2010/14 | 1,101 | 24.7 (22.1–27.3) | **24.5** | **24.8** | **23.5** | **29.6** | NA | NA | unweighted |
| **Egypt** | **WVS** | **2010/14** | **1,101** | **26.4 (22.3–30.5)** | **23.9** | **28.9** | **25.2** | **33.3** | **NA** | **NA** | **weighted** |
| Egypt | WVS | 2017/21 | 890 | 41.0 (37.8–44.2) | 40.4 | 41.6 | 45.2 | 50 | 44.9 | 37.8 | unweighted |
| **Egypt** | **WVS** | **2017/21** | **890** | **41.0 (37.7–44.2)** | **40.4** | **41.6** | **45.2** | **50** | **44.9** | **37.8** | **weighted** |
| Estonia | WVS | 2010/14 | 1,218 | 12.3 (10.4–14.1) | **12.3** | **12.3** | **14** | **10.6** | NA | NA | unweighted |
| **Estonia** | **WVS** | **2010/14** | **1,218** | **12.0 (10.2–13.9)** | **12.1** | **12** | **14** | **10.5** | **NA** | **NA** | **weighted** |
| Ethiopia | WVS | 2017/21 | 603 | 20.8 (17.5–24.0) | 18.4 | 23.7 | 24.3 | 33.3 | 13.8 | 23.1 | unweighted |
| **Ethiopia** | **WVS** | **2017/21** | **603** | **20.8 (17.5–24.0)** | **18.4** | **23.7** | **24.3** | **33.3** | **13.8** | **23.1** | **weighted** |
| France | CMWF | 2017 | 77 | 10.3 (8.1–12.4) | 8.7 | 11.5 | 11.1 | 9.8 | 10.0 | 10.4 | unweighted |
| **France** | **CMWF** | **2017** | **77** | **9.5 (7.3–11.7)** | **7.5** | **11.1** | **9.8** | **9.4** | **8.4** | **10.1** | **weighted** |
| Gambia | IHS | 2015 | 514 | 20.3 (18.8–21.9) | 22.1 | 19.2 | 19.2 | 23.6 | 21.0 | 20.3 | unweighted |
| Georgia | WVS | 2010/14 | 904 | 39.5 (36.3–42.7) | **35.7** | **42.7** | **47.7** | **52.7** | NA | NA | unweighted |
| **Georgia** | **WVS** | **2010/14** | **904** | **39.5 (36.3–42.7)** | **35.7** | **42.7** | **47.7** | **52.7** | **NA** | **NA** | **weighted** |
| Germany | CMWF | 2017 | 97 | 13.0 (10.6–15.4) | 14.0 | 12.1 | 13.3 | 12.9 | 11.9 | 14.5 | unweighted |
| **Germany** | **CMWF** | **2017** | **97** | **13.1 (10.4–15.9)** | **13.6** | **12.7** | **12.8** | **13.3** | **12.9** | **13.5** | **weighted** |
| Germany | WVS | 2010/14 | 1,691 | 2.7 (1.9–3.5) | **2.7** | **2.8** | **1.4** | **2.8** | NA | NA | unweighted |
| **Germany** | **WVS** | **2010/14** | **1,691** | **3.6 (1.8–5.5)** | **4.4** | **2.9** | **0.7** | **2** | **NA** | **NA** | **weighted** |
| Germany | WVS | 2017/21 | 1,280 | 4.5 (3.4–5.7) | 3.4 | 5.6 | 3 | 6.2 | 4.3 | 6.3 | unweighted |
| **Germany** | **WVS** | **2017/21** | **1,280** | **4.5 (3.4–5.7)** | **3.4** | **5.6** | **3** | **6.2** | **4.3** | **6.3** | **weighted** |
| Ghana | SAGE-W1 | 2007/08 | 1006 | 22.1 (20.9-23.3) | 23.2 | 21.0 | 23.1 | 23.9 | 17.8 | 25.2 | unweighted |
| **Ghana** | **SAGE-W1** | **2007/08** | **1006** | **17.6 (14.9-20.6)** | **19.9** | **15.2** | **21.4** | **22.6** | **14.1** | **20.5** | **weighted** |
| Ghana | SAGE-W2 | 2014/15 | 463 | 16.7 (15.3-18.1) | 17.1 | 16.4 | 18.8 | 18.1 | 15.5 | 17.6 | unweighted |
| **Ghana** | **SAGE-W2** | **2014/15** | **463** | **15.3 (12.6-18.4)** | **15.2** | **15.3** | **18.6** | **18.5** | **15.4** | **15.2** | **weighted** |
| Ghana | WVS | 2010/14 | 614 | 26.2 (22.7–29.7) | **28.5** | **24.2** | **34.5** | **36.8** | NA | NA | unweighted |
| **Ghana** | **WVS** | **2010/14** | **614** | **32.3 (27.9–36.7)** | **33.1** | **31.5** | **42.6** | **47.2** | **NA** | **NA** | **weighted** |
| Greece | WVS | 2017/21 | 1,044 | 6.9 (5.4–8.5) | 6.8 | 7.1 | 6.1 | 10.2 | 7.1 | 6.4 | unweighted |
| **Greece** | **WVS** | **2017/21** | **1,044** | **7.6 (5.7–9.4)** | **7** | **8** | **7** | **10.9** | **7.6** | **7.4** | **weighted** |
| Guatemala | WVS | 2017/21 | 614 | 21.8 (18.6–25.1) | 20.2 | 23 | 25 | 25.9 | 24.8 | 14.3 | unweighted |
| **Guatemala** | **WVS** | **2017/21** | **614** | **21.8 (18.5–25.1)** | **20.2** | **23** | **25** | **25.9** | **24.8** | **14.3** | **weighted** |
| Haiti | WVS | 2010/14 | 958 | 55.2 (52.0–58.4) | **55.6** | **54.8** | **52.1** | **47.6** | NA | NA | unweighted |
| **Haiti** | **WVS** | **2010/14** | **958** | **55.2 (52.0–58.4)** | **55.6** | **54.8** | **52.1** | **47.6** | **NA** | **NA** | **weighted** |
| Hong Kong SAR | WVS | 2010/14 | 796 | 8.7 (6.7–10.6) | **9** | **8.4** | **9.8** | **11.6** | NA | NA | unweighted |
| **Hong Kong SAR** | **WVS** | **2010/14** | **796** | **8.7 (6.7–10.6)** | **9** | **8.4** | **9.8** | **11.6** | **NA** | **NA** | **weighted** |
| Hong Kong SAR | WVS | 2017/21 | 1,746 | 12.2 (10.6–13.7) | 13.7 | 10.9 | 8.5 | 10.2 | 12.2 | 14.3 | unweighted |
| **Hong Kong SAR** | **WVS** | **2017/21** | **1,746** | **12.1 (10.4–13.7)** | **13.7** | **10.7** | **8.6** | **10.4** | **12.1** | **14.3** | **weighted** |
| India | SAGE-W1 | 2009 | 1621 | 18.6 (17.8-19.4) | 18.3 | 18.8 | 21.5 | 28.7 | 14.0 | 20.2 | unweighted |
| **India** | **SAGE-W1** | **2009** | **1621** | **14.4 (13.0-16.0)** | **13.0** | **16.0** | **21.5** | **27.2** | **12.9** | **15.0** | **weighted** |
| India | WVS | 2010/14 | 3,110 | 29.2 (27.6–30.8) | **28.6** | **29.9** | **30.4** | **28.4** | NA | NA | unweighted |
| **India** | **WVS** | **2010/14** | **3,110** | **29.2 (27.6–30.8)** | **28.6** | **29.9** | **30.4** | **28.4** | **NA** | **NA** | **weighted** |
| Indonesia | WVS | 2017/21 | 2,412 | 37.7 (35.7–39.6) | 38.7 | 36.7 | 45 | 38.5 | 31.2 | 39.9 | unweighted |
| **Indonesia** | **WVS** | **2017/21** | **2,412** | **36.9 (34.4–39.5)** | **38.3** | **35.6** | **42** | **37.6** | **32.5** | **38.7** | **weighted** |
| Iran (Islamic Republic of) | WVS | 2017/21 | 1,079 | 34.2 (31.4–37.1) | 35.2 | 33.2 | 31.2 | 34.7 | 33.6 | 36.3 | unweighted |
| **Iran (Islamic Republic of)** | **WVS** | **2017/21** | **1,079** | **34.2 (31.4–37.1)** | **35.2** | **33.2** | **31.2** | **34.7** | **33.6** | **36.3** | **weighted** |
| Iraq | WVS | 2010/14 | 761 | 19.8 (17.0–22.7) | **17.9** | **21.7** | **18.8** | **13.3** | NA | NA | unweighted |
| **Iraq** | **WVS** | **2010/14** | **761** | **19.8 (17.0–22.7)** | **17.9** | **21.7** | **18.8** | **13.3** | **NA** | **NA** | **weighted** |
| Iraq | WVS | 2017/21 | 733 | 38.9 (35.4–42.4) | 37.4 | 40.4 | 47.7 | 34.7 | 40 | 36.7 | unweighted |
| **Iraq** | **WVS** | **2017/21** | **733** | **38.9 (35.4–42.4)** | **37.4** | **40.4** | **47.7** | **34.7** | **40** | **36.7** | **weighted** |
| Japan | WVS | 2010/14 | 2,147 | 4.5 (3.6–5.4) | **5.1** | **3.9** | **4** | **3.7** | NA | NA | unweighted |
| **Japan** | **WVS** | **2010/14** | **2,147** | **4.5 (3.6–5.4)** | **5.1** | **3.9** | **4** | **3.7** | **NA** | **NA** | **weighted** |
| Japan | WVS | 2017/21 | 1,216 | 4.0 (2.9–5.1) | 2.8 | 5 | 4.1 | 5.1 | 4 | 4 | unweighted |
| **Japan** | **WVS** | **2017/21** | **1,216** | **4.0 (2.9–5.1)** | **2.8** | **5** | **4.1** | **5.1** | **4** | **4** | **weighted** |
| Jordan | WVS | 2010/14 | 852 | 17.6 (15.0–20.2) | **14** | **20.7** | **13.3** | **14.3** | NA | NA | unweighted |
| **Jordan** | **WVS** | **2010/14** | **852** | **17.6 (15.0–20.2)** | **14** | **20.7** | **13.3** | **14.3** | **NA** | **NA** | **weighted** |
| Jordan | WVS | 2017/21 | 941 | 21.2 (18.6–23.8) | 19.1 | 23 | 19.5 | 6.5 | 21 | 21.6 | unweighted |
| **Jordan** | **WVS** | **2017/21** | **941** | **21.2 (18.6–23.8)** | **19.1** | **23** | **19.5** | **6.5** | **21** | **21.6** | **weighted** |
| Kazakhstan | WVS | 2010/14 | 1,041 | 12.5 (10.5–14.5) | **14.2** | **11.4** | **15.6** | **15.2** | NA | NA | unweighted |
| **Kazakhstan** | **WVS** | **2010/14** | **1,041** | **12.6 (10.5–14.7)** | **13.8** | **11.6** | **15.4** | **15.8** | **NA** | **NA** | **weighted** |
| Kazakhstan | WVS | 2017/21 | 968 | 13.7 (11.5–15.9) | 13 | 14.2 | 22.2 | 15.9 | 14.2 | 13 | unweighted |
| **Kazakhstan** | **WVS** | **2017/21** | **968** | **13.7 (11.5–15.9)** | **13** | **14.2** | **22.2** | **15.9** | **14.2** | **13** | **weighted** |
| Kuwait | WVS | 2010/14 | 900 | 8.5 (6.6–10.3) | **8.6** | **8.2** | **7.7** | **5.9** | NA | NA | unweighted |
| **Kuwait** | **WVS** | **2010/14** | **900** | **8.5 (6.6–10.3)** | **8.6** | **8.2** | **7.7** | **5.9** | **NA** | **NA** | **weighted** |
| Kyrgyzstan | WVS | 2010/14 | 1,005 | 18.3 (15.8–20.8) | **17.4** | **19.1** | **13.7** | **17.8** | NA | NA | unweighted |
| **Kyrgyzstan** | **WVS** | **2010/14** | **1,005** | **18.3 (15.8–20.8)** | **17.4** | **19.1** | **13.7** | **17.8** | **NA** | **NA** | **weighted** |
| Kyrgyzstan | WVS | 2017/21 | 879 | 9.3 (7.3–11.2) | 9 | 9.4 | 12.4 | 6.8 | 12.9 | 7.2 | unweighted |
| **Kyrgyzstan** | **WVS** | **2017/21** | **879** | **8.7 (6.7–10.7)** | **8.3** | **9** | **11.3** | **5.6** | **11.8** | **6.9** | **weighted** |
| Lebanon | WVS | 2010/14 | 747 | 23.4 (20.3–26.5) | **23.5** | **23.3** | **24.7** | **25** | NA | NA | unweighted |
| **Lebanon** | **WVS** | **2010/14** | **747** | **23.4 (20.3–26.5)** | **23.5** | **23.3** | **24.7** | **25** | **NA** | **NA** | **weighted** |
| Lebanon | WVS | 2017/21 | 837 | 9.9 (7.9–11.9) | 10 | 9.8 | 14.4 | 10.7 | 10.5 | 4.6 | unweighted |
| **Lebanon** | **WVS** | **2017/21** | **837** | **9.9 (7.9–11.9)** | **10** | **9.8** | **14.4** | **10.7** | **10.5** | **4.6** | **weighted** |
| Libya | WVS | 2010/14 | 1,471 | 24.3 (22.1–26.5) | **29.2** | **20** | **20** | **21.7** | NA | NA | unweighted |
| **Libya** | **WVS** | **2010/14** | **1,471** | **24.8 (21.7–27.9)** | **30.2** | **19.3** | **18.5** | **15.8** | **NA** | **NA** | **weighted** |
| Macau SAR | WVS | 2017/21 | 735 | 6.3 (4.5–8.1) | 6.3 | 6.3 | 8.7 | 2.3 | 6.3 | 4.6 | unweighted |
| **Macau SAR** | **WVS** | **2017/21** | **735** | **6.3 (4.5–8.1)** | **6.3** | **6.3** | **8.7** | **2.3** | **6.3** | **4.6** | **weighted** |
| Malaysia | WVS | 2010/14 | 940 | 5.8 (4.3–7.2) | **8.4** | **3** | **6.7** | **14.7** | NA | NA | unweighted |
| **Malaysia** | **WVS** | **2010/14** | **940** | **5.8 (4.3–7.2)** | **8.4** | **3** | **6.7** | **14.7** | **NA** | **NA** | **weighted** |
| Malaysia | WVS | 2017/21 | 894 | 15.3 (13.0–17.7) | 14.3 | 16.4 | 11.1 | 8.3 | 20.4 | 6 | unweighted |
| **Malaysia** | **WVS** | **2017/21** | **894** | **15.3 (13.0–17.7)** | **14.3** | **16.4** | **11.1** | **8.3** | **20.4** | **6** | **weighted** |
| Mexico | MHAS | 2001 | 2,286 | 18.7 (18.0-19.4) | 15.5 | 21.4 | 18.9 | 17.6 | 17.2 | 21.6 | unweighted |
| **Mexico** | **MHAS** | **2001** | **2,286** | **20.2 (18.8-21.6)** | **16.8** | **23.0** | **20.0** | **18.7** | **17.2** | **22.7** | **weighted** |
| Mexico | MHAS | 2003 | 2,495 | 21.6 (20.9-22.4) | 17.6 | 24.7 | 20.9 | 20.5 | N/A | N/A | unweighted |
| **Mexico** | **MHAS** | **2003** | **2,495** | **23.3 (21.8-24.8)** | **19.7** | **26.2** | **23.2** | **21.8** | **N/A** | **N/A** | **weighted** |
| Mexico | MHAS | 2012 | 2,520 | 18.5 (17.9-19.2) | 15.5 | 20.7 | 18.8 | 16.2 | 16.6 | 21.4 | unweighted |
| **Mexico** | **MHAS** | **2012** | **2,520** | **20.9 (19.3-22.6)** | **17.0** | **24.2** | **21.7** | **17.4** | **19.0** | **22.7** | **weighted** |
| Mexico | MHAS | 2015 | 2,359 | 17.8 (17.2-18.5) | 14.8 | 20.0 | 17.8 | 15.6 | 15.8 | 20.6 | unweighted |
| **Mexico** | **MHAS** | **2015** | **2,359** | **19.8 (18.3-21.3)** | **16.4** | **22.5** | **20.5** | **16.4** | **17.4** | **22.1** | **weighted** |
| Mexico | MHAS | 2018 | 2,547 | 17.2 (16.6-17.8) | 14.5 | 19.3 | 17.1 | 14.5 | 15.4 | 19.6 | unweighted |
| **Mexico** | **MHAS** | **2018** | **2,547** | **18.5 (17.3-19.8)** | **15.2** | **21.0** | **18.0** | **17.5** | **14.4** | **22.2** | **weighted** |
| Mexico | SAGE-W1 | 2010 | 403 | 17.7 (16.1-19.2) | 17.4 | 17.8 | 16.7 | 21.7 | 17.4 | 18.3 | unweighted |
| **Mexico** | **SAGE-W1** | **2010** | **403** | **13.5 (9.5-18.7)** | **15.0** | **12.3** | **17.7** | **21.8** | **14.6** | **9.8** | **weighted** |
| Mexico | SAGE-W2 | 2014 | 724 | 18.2 (17.0-19.4) | 17.7 | 18.6 | 18.5 | 18.2 | 17.9 | 19.2 | unweighted |
| **Mexico** | **SAGE-W2** | **2014** | **724** | **18.1 (14.2-22.6)** | **17.5** | **18.5** | **17.5** | **17.9** | **18.0** | **18.3** | **weighted** |
| Mexico | WVS | 2010/14 | 1,217 | 33.6 (31.0–36.3) | **32.6** | **34.7** | **27.8** | **22.8** | NA | NA | unweighted |
| **Mexico** | **WVS** | **2010/14** | **1,217** | **33.6 (30.9–36.3)** | **32.6** | **34.7** | **27.8** | **22.8** | **NA** | **NA** | **weighted** |
| Mexico | WVS | 2017/21 | 1,296 | 29.1 (26.6–31.6) | 26.6 | 31.6 | 30 | 21.6 | 28.2 | 30.9 | unweighted |
| **Mexico** | **WVS** | **2017/21** | **1,296** | **29.1 (26.6–31.6)** | **26.5** | **31.4** | **29.4** | **21.7** | **28.1** | **31.1** | **weighted** |
| Mongolia | MiniSAGE | 2017 | 194 | 22.1 (19.4-24.9) | 22.7 | 21.8 | 21.5 | 23.2 | 13.8 | 30.4 | unweighted |
| Morocco | WVS | 2010/14 | 709 | 55.2 (51.5–58.9) | **54** | **56.4** | **55.4** | **44.4** | NA | NA | unweighted |
| **Morocco** | **WVS** | **2010/14** | **709** | **55.2 (51.5–58.9)** | **54** | **56.4** | **55.4** | **44.4** | **NA** | **NA** | **weighted** |
| Myanmar | MAS | 2012 | 268 | 30.0 (27.6–32.4) | 28.3 | 31.0 | 31.2 | 29.1 | 29.1 | 30.3 | unweighted |
| **Myanmar** | **MAS** | **2012** | **268** | **29.1 (26.6–31.7)** | **26.9** | **30.8** | **29.8** | **28.5** | **26.7** | **30.2** | **weighted** |
| Myanmar | Survey on Accessing Healthcare to Older Population | 2016 | 10 | 1.6 (0.8–3.0) | 0.4 | 2.5 | 1.7 | 1.5 | 2.0 | 1.4 | unweighted |
| Myanmar | WVS | 2017/21 | 874 | 11.4 (9.3–13.6) | 13 | 9.8 | 15.6 | 27.3 | 8.6 | 12.7 | unweighted |
| **Myanmar** | **WVS** | **2017/21** | **874** | **11.4 (9.3–13.6)** | **13** | **9.8** | **15.6** | **27.3** | **8.6** | **12.7** | **weighted** |
| Netherlands | CMWF | 2017 | 70 | 9.3 (7.3–11.4) | 9.4 | 9.2 | 12.1 | 8.1 | NA | NA | unweighted |
| **Netherlands** | **CMWF** | **2017** | **70** | **9.7 (7.3–12.0)** | **8.8** | **10.3** | **12.2** | **8.4** | **NA** | **NA** | **weighted** |
| Netherlands | WVS | 2010/14 | 1,712 | 1.6 (1.0–2.2) | **1.7** | **1.5** | **1.1** | **1.6** | NA | NA | unweighted |
| **Netherlands** | **WVS** | **2010/14** | **1,712** | **1.6 (1.0–2.2)** | **1.7** | **1.5** | **1.1** | **1.6** | **NA** | **NA** | **weighted** |
| New Zealand | CMWF | 2017 | 116 | 23.2 (19.5–26.9) | 23.7 | 22.8 | 28.6 | 18.6 | NA | NA | unweighted |
| **New Zealand** | **CMWF** | **2017** | **116** | **22.0 (17.9–26.1)** | **22.2** | **21.9** | **32.4** | **17.0** | **NA** | **NA** | **weighted** |
| **New Zealand** | **HS** | **2014/15** | **1,917** | **17.6 (17.5-17.6)** | **14.9** | **20.0** | **14.3** | **12.0** | **17.8** | **15.9** | **weighted** |
| New Zealand | HS | 2014/15 | 1,917 | 18.5 (17.8-19.3) | 16.5 | 20.0 | 14.9 | 12.6 | 18.7 | 17.0 | unweighted |
| **New Zealand** | **HS** | **2015/16** | **2030** | **18.7 (18.7-18.8)** | **15.0** | **21.6** | **16.2** | **12.1** | **18.8** | **15.9** | **weighted** |
| New Zealand | HS | 2015/16 | 2030 | 19.1 (18.4-19.8) | 15.3 | 21.9 | 17.0 | 12.3 | 19.3 | 17.4 | unweighted |
| **New Zealand** | **HS** | **2016/17** | **2116** | **18.5 (18.4-18.5)** | **15.9** | **21.2** | **17.3** | **14.6** | **18.7** | **18.5** | **weighted** |
| New Zealand | HS | 2016/17 | 2116 | 20.0 (19.2-20.7) | 17.0 | 22.1 | 18.3 | 14.0 | 19.8 | 21.0 | unweighted |
| **New Zealand** | **HS** | **2017/18** | **2355** | **20.5 (20.4-20.5)** | **16.7** | **23.8** | **17.5** | **13.0** | **20.8** | **19.0** | **weighted** |
| New Zealand | HS | 2017/18 | 2355 | 21.8 (21.0-22.6) | 17.4 | 25.0 | 19.1 | 13.8 | 22.1 | 20.3 | unweighted |
| **New Zealand** | **HS** | **2018/19** | **2409** | **21.3 (21.3-21.4)** | **18.0** | **24.3** | **18.1** | **15.3** | **21.0** | **22.9** | **weighted** |
| New Zealand | HS | 2018/19 | 2409 | 22.5 (21.7-23.3) | 18.3 | 25.3 | 20.0 | 16.1 | 22.1 | 24.6 | unweighted |
| New Zealand | WVS | 2010/14 | 741 | 11.7 (9.4–14.1) | **13.3** | **10.7** | **7.5** | **6.6** | NA | NA | unweighted |
| **New Zealand** | **WVS** | **2010/14** | **741** | **11.7 (9.4–14.1)** | **13.3** | **10.7** | **7.5** | **6.6** | **NA** | **NA** | **weighted** |
| New Zealand | WVS | 2017/21 | 994 | 9.8 (7.9–11.6) | 9.2 | 10 | 8.3 | 7.9 | 9.5 | 12 | unweighted |
| **New Zealand** | **WVS** | **2017/21** | **994** | **9.8 (7.9–11.6)** | **9.2** | **10** | **8.3** | **7.9** | **9.5** | **12** | **weighted** |
| Nicaragua | WVS | 2017/21 | 692 | 48.4 (44.7–52.1) | 48.7 | 48.2 | 45.2 | 41.7 | 44.8 | 52.5 | unweighted |
| **Nicaragua** | **WVS** | **2017/21** | **692** | **48.4 (44.7–52.1)** | **48.7** | **48.2** | **45.2** | **41.7** | **44.8** | **52.5** | **weighted** |
| Nigeria | WVS | 2010/14 | 767 | 33.2 (29.8–36.6) | **34.8** | **31.2** | **41.5** | **33.3** | NA | NA | unweighted |
| **Nigeria** | **WVS** | **2010/14** | **767** | **34.1 (29.9–38.2)** | **34.5** | **33.5** | **45.6** | **28** | **NA** | **NA** | **weighted** |
| Nigeria | WVS | 2017/21 | 622 | 44.4 (40.5–48.4) | 44.8 | 44 | 34.9 | 46.7 | 42.6 | 46.3 | unweighted |
| **Nigeria** | **WVS** | **2017/21** | **622** | **44.3 (40.3–48.3)** | **44.4** | **44.2** | **34.9** | **46.7** | **42.6** | **46.1** | **weighted** |
| Norway | CMWF | 2017 | 65 | 8.7 (6.6–10.7) | 8.5 | 8.8 | 12.2 | 7.0 | 7.4 | 10.6 | unweighted |
| **Norway** | **CMWF** | **2017** | **65** | **9.9 (7.3–12.4)** | **8.5** | **11.0** | **12.5** | **8.7** | **8.5** | **11.8** | weighted |
| Pakistan | WVS | 2010/14 | 744 | 37.1 (33.6–40.6) | **39** | **35** | **42.9** | **23.1** | NA | NA | unweighted |
| **Pakistan** | **WVS** | **2010/14** | **744** | **37.0 (33.4–40.6)** | **38.2** | **35.7** | **41.7** | **22.8** | **NA** | **NA** | **weighted** |
| Pakistan | WVS | 2017/21 | 1,367 | 43.1 (40.5–45.8) | 41.5 | 45 | 45.3 | 57.9 | 38.7 | 45.3 | unweighted |
| **Pakistan** | **WVS** | **2017/21** | **1,367** | **43.1 (40.5–45.8)** | **41.5** | **45** | **45.3** | **57.9** | **38.7** | **45.3** | **weighted** |
| Palestine | WVS | 2010/14 | 618 | 22.3 (19.0–25.6) | **22.3** | **22.4** | **27.5** | **32.3** | NA | NA | unweighted |
| **Palestine** | **WVS** | **2010/14** | **618** | **22.3 (19.0–25.6)** | **22.3** | **22.4** | **27.5** | **32.3** | **NA** | **NA** | **weighted** |
| Peru | WVS | 2010/14 | 800 | 37.7 (34.3–41.1) | **35.5** | **39.7** | **40.9** | **37.7** | NA | NA | unweighted |
| **Peru** | **WVS** | **2010/14** | **800** | **37.5 (34.1–40.9)** | **35.1** | **39.9** | **41.1** | **37.9** | **NA** | **NA** | **weighted** |
| Peru | WVS | 2017/21 | 964 | 39.6 (36.5–42.7) | 39 | 40.1 | 45.2 | 31.7 | 36.6 | 49.5 | unweighted |
| **Peru** | **WVS** | **2017/21** | **964** | **39.5 (36.1–42.8)** | **38.4** | **40.5** | **47.5** | **29.7** | **37.3** | **48.3** | **weighted** |
| Philippines | WVS | 2010/14 | 918 | 33.6 (30.5–36.6) | **35** | **32.1** | **24.4** | **31.8** | NA | NA | unweighted |
| **Philippines** | **WVS** | **2010/14** | **918** | **34.5 (31.1–37.9)** | **35.5** | **33.5** | **22.7** | **31.1** | **NA** | **NA** | **weighted** |
| Philippines | WVS | 2017/21 | 927 | 29.9 (26.9–32.8) | 27.2 | 32.5 | 42.1 | 30.1 | 28.4 | 31.7 | unweighted |
| **Philippines** | **WVS** | **2017/21** | **927** | **28.2 (25.0–31.3)** | **26** | **30.3** | **38** | **29.8** | **26.8** | **29.5** | **weighted** |
| Poland | WVS | 2010/14 | 766 | 12.4 (10.1–14.8) | **13** | **12** | **8.5** | **20** | NA | NA | unweighted |
| **Poland** | **WVS** | **2010/14** | **766** | **12.5 (10.0–15.0)** | **12.5** | **12.6** | **6.9** | **21.2** | **NA** | **NA** | **weighted** |
| **Puerto Rico** | **PREHCO W1** | **2002/3** | **872** | **22.0 (19.2 - 25.0)** | **16.1** | **26.3** | **23.8** | **20.1** | **NA** | **NA** | **weighted** |
| **Puerto Rico** | **PREHCO W2** | **2006/7** | **499** | **17.5 (15.3 - 19.8)** | **15.2** | **19.1** | **18.8** | **16.7** | **NA** | **NA** | **weighted** |
| Puerto Rico | WVS | 2017/21 | 921 | 18.7 (16.2–21.2) | 20.1 | 17.9 | 17.3 | 10.4 | 18.3 | 25.4 | unweighted |
| **Puerto Rico** | **WVS** | **2017/21** | **921** | **18.7 (16.2–21.2)** | **20.1** | **17.9** | **17.3** | **10.4** | **18.3** | **25.4** | **weighted** |
| Qatar | WVS | 2010/14 | 747 | 0.9 (0.2–1.6) | **0.9** | **1** | **0** | **0** | NA | NA | unweighted |
| **Qatar** | **WVS** | **2010/14** | **747** | **0.9 (0.2–1.6)** | **0.8** | **1** | **0** | **0** | **NA** | **NA** | **weighted** |
| Republic of Korea | WVS | 2010/14 | 950 | 2.6 (1.6–3.6) | **2.9** | **2.3** | **3.7** | **7.7** | NA | NA | unweighted |
| **Republic of Korea** | **WVS** | **2010/14** | **950** | **3.8 (1.7–5.9)** | **4.7** | **2.9** | **4.9** | **10.5** | **NA** | **NA** | **weighted** |
| Republic of Korea | WVS | 2017/21 | 997 | 2.0 (1.1–2.9) | 2.5 | 1.5 | 0.9 | 0 | 2 | 0 | unweighted |
| **Republic of Korea** | **WVS** | **2017/21** | **997** | **2.0 (1.1–2.9)** | **2.5** | **1.5** | **0.9** | **0** | **2** | **0** | **weighted** |
| Romania | WVS | 2010/14 | 1,247 | 32.3 (29.7–34.9) | **25.1** | **37.6** | **38.7** | **36** | NA | NA | unweighted |
| **Romania** | **WVS** | **2010/14** | **1,247** | **31.1 (28.5–33.8)** | **25.3** | **36.3** | **38** | **36.7** | **NA** | **NA** | **weighted** |
| Romania | WVS | 2017/21 | 1,009 | 15.5 (13.3–17.7) | 10.4 | 18.8 | 19.6 | 22.9 | 13.7 | 17.7 | unweighted |
| **Romania** | **WVS** | **2017/21** | **1,009** | **16.6 (14.0–19.3)** | **11.8** | **20.8** | **21.2** | **26** | **14.8** | **19.1** | **weighted** |
| Russian Federation | SAGE-W1 | 2010 | 1044 | 29.3 (27.8-30.8) | 27.4 | 30.3 | 26.8 | 26.0 | 29.6 | 28.4 | unweighted |
| **Russian Federation** | **SAGE-W1** | **2010** | **1044** | **43.6 (33.1-54.8)** | **46.5** | **42.1** | **20.0** | **18.0** | **47.9** | **27.4** | **weighted** |
| Russian Federation | WVS | 2010/14 | 1,933 | 16.1 (14.4–17.7) | **14.2** | **17.4** | **20.6** | **20.9** | NA | NA | unweighted |
| **Russian Federation** | **WVS** | **2010/14** | **1,933** | **15.9 (14.2–17.6)** | **14.4** | **16.9** | **19.6** | **20.7** | **NA** | **NA** | **weighted** |
| Russian Federation | WVS | 2017/21 | 1,425 | 10.2 (8.6–11.7) | 10.7 | 9.8 | 11.4 | 19.7 | 8.6 | 14.2 | unweighted |
| **Russian Federation** | **WVS** | **2017/21** | **1,425** | **10.3 (8.7–11.9)** | **10.8** | **9.9** | **11.5** | **19.8** | **8.6** | **14.4** | **weighted** |
| Rwanda | WVS | 2010/14 | 905 | 65.7 (62.6–68.8) | **64.8** | **66.7** | **68.4** | **59.1** | NA | NA | unweighted |
| **Rwanda** | **WVS** | **2010/14** | **905** | **65.7 (62.6–68.8)** | **64.8** | **66.7** | **68.4** | **59.1** | **NA** | **NA** | **weighted** |
| Serbia | WVS | 2017/21 | 815 | 13.5 (11.1–15.9) | 12.5 | 14.4 | 19.8 | 16.2 | 14.1 | 12.3 | unweighted |
| **Serbia** | **WVS** | **2017/21** | **815** | **13.5 (11.1–15.9)** | **12.5** | **14.4** | **19.8** | **16.2** | **14.1** | **12.3** | **weighted** |
| Singapore | WVS | 2010/14 | 1,398 | 6.6 (5.3–7.9) | **7.9** | **5.6** | **6.3** | **1.6** | NA | NA | unweighted |
| **Singapore** | **WVS** | **2010/14** | **1,398** | **6.3 (5.0–7.7)** | **7.5** | **5.5** | **6.1** | **1.7** | **NA** | **NA** | **weighted** |
| Slovenia | WVS | 2010/14 | 895 | 1.1 (0.4–1.8) | **0.8** | **1.4** | **2.1** | **0.6** | NA | NA | unweighted |
| **Slovenia** | **WVS** | **2010/14** | **895** | **1.1 (0.4–1.8)** | **0.8** | **1.4** | **2.1** | **0.6** | **NA** | **NA** | **weighted** |
| South Africa | SAGE-W1 | 2007/08 | 133 | 4.0 (3.3-4.7) | 4.5 | 3.7 | 4.3 | 3.8 | 3.2 | 6.0 | unweighted |
| **South Africa** | **SAGE-W1** | **2007/08** | **133** | **4.2 (3.5-4.8)** | **6.3** | **2.3** | **5.9** | **5.6** | **4.0** | **4.6** | **weighted** |
| South Africa | SAGE-W2 | 2014/15 | 262 | 24.8 (22.2-27.4) | 23.0 | 25.5 | 22.2 | 29.8 | 21.8 | 32.4 | unweighted |
| **South Africa** | **SAGE-W2** | **2014/15** | **262** | **22.4 (19.8-24.9)** | **21.2** | **23.1** | **25.9** | **29.2** | **22.7** | **21.1** | **weighted** |
| South Africa | WVS | 2010/14 | 2,213 | 32.8 (30.8–34.7) | **32.5** | **33** | **37.8** | **32.6** | NA | NA | unweighted |
| **South Africa** | **WVS** | **2010/14** | **2,213** | **34.8 (32.4–37.2)** | **35.5** | **34.2** | **37.7** | **33.3** | **NA** | **NA** | **weighted** |
| Spain | WVS | 2010/14 | 955 | 4.0 (2.7–5.2) | **4.3** | **3.7** | **5.6** | **1.8** | NA | NA | unweighted |
| **Spain** | **WVS** | **2010/14** | **955** | **3.9 (2.7–5.2)** | **4.2** | **3.7** | **5.5** | **1.8** | **NA** | **NA** | **weighted** |
| Sweden | CMWF | 2017 | 544 | 8.0 (7.4–8.7) | 7.6 | 7.9 | 8.6 | 7.6 | NA | NA | unweighted |
| **Sweden** | **CMWF** | **2017** | **544** | **8.5 (7.7–9.4)** | **7.7** | **8.7** | **8.7** | **8.1** | **NA** | **NA** | **weighted** |
| Sweden | WVS | 2010/14 | 901 | 5.3 (3.8–6.7) | **4.4** | **6** | **4.3** | **6.9** | NA | NA | unweighted |
| **Sweden** | **WVS** | **2010/14** | **901** | **5.3 (3.6–7.0)** | **3.9** | **6.7** | **4.4** | **6.6** | **NA** | **NA** | **weighted** |
| Switzerland | CMWF | 2017 | 668 | 21.0 (19.6–22.4) | 21.2 | 20.1 | 25.4 | 19.0 | NA | NA | unweighted |
| **Switzerland** | **CMWF** | **2017** | **668** | **19.4 (17.4–21.3)** | **21.2** | **16.8** | **21.1** | **17.8** | **NA** | **NA** | **weighted** |
| Taiwan ROC | WVS | 2010/14 | 950 | 7.5 (5.8–9.2) | **7.6** | **7.3** | **6.3** | **8.3** | NA | NA | unweighted |
| **Taiwan ROC** | **WVS** | **2010/14** | **950** | **8.0 (6.1–10.0)** | **8.1** | **8** | **6.2** | **6.9** | **NA** | **NA** | **weighted** |
| Taiwan ROC | WVS | 2017/21 | 1,018 | 5.5 (4.1–6.9) | 6.9 | 4.2 | 4.1 | 8.6 | 5.5 | 5.2 | unweighted |
| **Taiwan ROC** | **WVS** | **2017/21** | **1,018** | **5.7 (3.8–7.7)** | **6.5** | **5** | **3.1** | **9.9** | **5.6** | **6.7** | **weighted** |
| Tajikistan | WVS | 2017/21 | 852 | 15.0 (12.6–17.4) | 12.1 | 17.5 | 16.2 | 9.8 | 13 | 15.7 | unweighted |
| **Tajikistan** | **WVS** | **2017/21** | **852** | **15.0 (12.6–17.4)** | **12.1** | **17.5** | **16.2** | **9.8** | **13** | **15.7** | **weighted** |
| Thailand | HWS | 2011 | 1059 | 2.38 (2.24-2.52) | 2.2 | 2.5 | 2.8 | 2.9 | 2.1 | 2.7 | unweighted |
| **Thailand** | **HWS** | **2011** | **1059** | **2.80 (2.80-2.81)** | **2.6** | **3** | **3.4** | **3.3** | **2.7** | **2.8** | **weighted** |
| Thailand | HWS | 2013 | 1441 | 3.17 (3.01-3.33) | 3.2 | 3.1 | 3.6 | 4 | 2.9 | 3.5 | unweighted |
| **Thailand** | **HWS** | **2013** | **1441** | **3.33 (3.33-3.34)** | **3.3** | **3.4** | **3.9** | **4.8** | **3.4** | **3.3** | **weighted** |
| Thailand | HWS | 2015 | 2471 | 2.72 (2.62-2.83) | 2.8 | 2.6 | 3.3 | 3.4 | 2.6 | 2.9 | unweighted |
| **Thailand** | **HWS** | **2015** | **2471** | **3.08 (3.07-3.08)** | **3.2** | **3** | **3.9** | **4.1** | **3.1** | **3** | **weighted** |
| Thailand | HWS | 2017 | 703 | 1.60 (1.48-1.72) | 1.7 | 1.5 | 1.7 | 2.2 | 1.4 | 1.9 | unweighted |
| **Thailand** | **HWS** | **2017** | **703** | **1.82 (1.82-1.83)** | **1.9** | **1.7** | **2** | **2.5** | **1.6** | **2** | **weighted** |
| Thailand | WVS | 2010/14 | 1,055 | 17.4 (15.0–19.7) | **16** | **18.8** | **19** | **11.8** | NA | NA | unweighted |
| **Thailand** | **WVS** | **2010/14** | **1,055** | **17.3 (15.0–19.7)** | **15.9** | **18.8** | **19** | **11.8** | **NA** | **NA** | **weighted** |
| Thailand | WVS | 2017/21 | 1,309 | 25.8 (23.4–28.2) | 28 | 23.9 | 21.3 | 11.1 | 20.5 | 28.9 | unweighted |
| **Thailand** | **WVS** | **2017/21** | **1,309** | **25.7 (23.3–28.1)** | **27.6** | **24.1** | **21.3** | **11.1** | **20.8** | **28.6** | **weighted** |
| Timor-Leste | SHIFT | 2014/15 | 1712 (households) | 4.2 (3.6–5.5) | 0.0 | 0.0 | 0.0 | 0.0 | 22.1 | 77.9 | unweighted |
| Trinidad and Tobago | WVS | 2010/14 | 758 | 13.1 (10.7–15.5) | **10.9** | **14.9** | **13.5** | **11.2** | NA | NA | unweighted |
| **Trinidad and Tobago** | **WVS** | **2010/14** | **758** | **13.1 (10.7–15.5)** | **10.9** | **14.9** | **13.5** | **11.2** | **NA** | **NA** | **weighted** |
| Tunisia | THES | 2016 | 2074 | 30.9 (29.8-32.0) | 30.0 | 31.7 | 32.1 | 32.4 | 30.4 | 31.9 | unweighted |
| **Tunisia** | **THES** | **2016** | **2074** | **30.1 (28.2-32.0)** | **28.9** | **31.2** | **31.4** | **31.0** | **29.6** | **31.2** | **weighted** |
| Tunisia | WHS | 2003 | 199 | 8.0 (6.9-9.0) | 8.3 | 7.0 | 16.0 | 23.2 | 6.4 | 10.5 | unweighted |
| **Tunisia** | **WHS** | **2003** | **199** | **7.8 (6.5-9.3)** | **7.3** | **8.4** | **17.8** | **24.9** | **6.5** | **10.1** | **weighted** |
| Tunisia | WVS | 2010/14 | 747 | 14.6 (12.0–17.1) | **13.4** | **15.8** | **24.2** | **23.1** | NA | NA | unweighted |
| **Tunisia** | **WVS** | **2010/14** | **747** | **14.6 (12.0–17.1)** | **13.4** | **15.8** | **24.2** | **23.1** | **NA** | **NA** | **weighted** |
| Tunisia | WVS | 2017/21 | 922 | 35.0 (31.9–38.1) | 37.1 | 33.5 | 38.2 | 38.7 | 30.2 | 44.9 | unweighted |
| **Tunisia** | **WVS** | **2017/21** | **922** | **35.0 (31.9–38.1)** | **37.1** | **33.5** | **38.2** | **38.7** | **30.2** | **44.9** | **weighted** |
| Turkey | WVS | 2010/14 | 1,094 | 12.9 (10.9–14.9) | **13.8** | **12.1** | **11.4** | **7.4** | NA | NA | unweighted |
| **Turkey** | **WVS** | **2010/14** | **1,094** | **13.8 (11.6–16.1)** | **15.3** | **12.4** | **11.3** | **8.1** | **NA** | **NA** | **weighted** |
| Turkey | WVS | 2017/21 | 1,703 | 17.3 (15.5–19.1) | 15 | 19.6 | 10 | 25 | 17.2 | 17.7 | unweighted |
| **Turkey** | **WVS** | **2017/21** | **1,703** | **17.3 (15.5–19.1)** | **15** | **19.6** | **10** | **25** | **17.2** | **17.7** | **weighted** |
| Ukraine | WVS | 2010/14 | 1,163 | 24.7 (22.2–27.3) | **23.4** | **25.5** | **24.7** | **40.7** | NA | NA | unweighted |
| **Ukraine** | **WVS** | **2010/14** | **1,163** | **23.9 (21.4–26.5)** | **21.9** | **25.5** | **25.1** | **41.5** | **NA** | **NA** | **weighted** |
| Ukraine | WVS | 2017/21 | 1,100 | 16.2 (14.0–18.4) | 13.6 | 17.9 | 21.6 | 29.7 | 15 | 18.6 | unweighted |
| **Ukraine** | **WVS** | **2017/21** | **1,100** | **16.2 (14.0–18.4)** | **13.8** | **17.8** | **21.3** | **29.1** | **15** | **18.7** | **weighted** |
| United Kingdom of Great Britain and Northern Ireland | CMWF | 2017 | 76 | 10.1 (7.9–12.2) | 11.0 | 9.4 | 9.3 | 10.0 | 9.8 | 9.1 | unweighted |
| **United Kingdom of Great Britain and Northern Ireland** | **CMWF** | **2017** | **76** | **10.2 (7.7–12.6)** | **11.2** | **8.9** | **10.0** | **10.0** | **10.5** | **8.5** | **weighted** |
| United States of America | CMWF | 2017 | 382 | 27.5 (25.1–29.8) | 25.6 | 28.6 | 37.8 | 24.6 | NA | NA | unweighted |
| **United States of America** | **CMWF** | **2017** | **382** | **32.7 (29.4–36.0)** | **29.1** | **35.5** | **42.7** | **27.8** | **NA** | **NA** | **weighted** |
| United States of America | WVS | 2010/14 | 1,839 | 17.0 (15.2–18.7) | **12.7** | **20.9** | **14.1** | **4.8** | NA | NA | unweighted |
| **United States of America** | **WVS** | **2010/14** | **1,839** | **19.5 (17.3–21.8)** | **14.1** | **24.5** | **12.4** | **6.9** | **NA** | **NA** | **weighted** |
| United States of America | WVS | 2017/21 | 1,964 | 17.6 (15.9–19.3) | 14.9 | 21.5 | 12.6 | 5.9 | 17.4 | 19.7 | unweighted |
| **United States of America** | **WVS** | **2017/21** | **1,964** | **16.6 (14.6–18.6)** | **14.7** | **18.7** | **10.1** | **7.9** | **16.3** | **19.2** | **weighted** |
| Uruguay | WVS | 2010/14 | 745 | 17.7 (14.9–20.4) | **13.9** | **20.9** | **8.5** | **19.3** | NA | NA | unweighted |
| **Uruguay** | **WVS** | **2010/14** | **745** | **17.7 (14.9–20.4)** | **13.9** | **20.9** | **8.5** | **19.3** | **NA** | **NA** | **weighted** |
| Uzbekistan | WVS | 2010/14 | 1,056 | 17.6 (15.3–19.9) | **17.5** | **17.7** | **16.2** | **14.3** | NA | NA | unweighted |
| **Uzbekistan** | **WVS** | **2010/14** | **1,056** | **17.6 (15.3–19.9)** | **17.5** | **17.7** | **16.2** | **14.3** | **NA** | **NA** | **weighted** |
| **Viet Nam** | **VNAS** | **2011** | **1012** | **5.4 (3.6–7.9)** | **4.0** | **6.3** | **5.4** | **5.3** | **6.2** | **5.0** | **weighted** |
| **Viet Nam** | **OP&SHI** | **2019** | **758** | **0.3 (0.1–0.9)** | **0.1** | **0.4** | **0.2** | **0.4** | **0.0** | **0.4** | **weighted** |
| Viet Nam | VNAS | 2011 | 1012 | 5.4 (3.6–7.9) | N/A | N/A | N/A | N/A | 2.9 | 7.2 | *0.4643* |
| Viet Nam | OP&SHI | 2019 | 758 | 0.3 (0.1–0.9) | N/A | N/A | N/A | N/A | 1.5 | 0.6 | *0.9194* |
| Viet Nam | WVS | 2017/21 | 832 | 9.0 (7.1–11.0) | 8.1 | 9.7 | 13.4 | 16.7 | 10.7 | 8.1 | unweighted |
| **Viet Nam** | **WVS** | **2017/21** | **832** | **9.0 (7.1–11.0)** | **8.1** | **9.7** | **13.4** | **16.7** | **10.7** | **8.1** | **weighted** |
| Yemen | WVS | 2010/14 | 639 | 39.2 (35.4–43.0) | **39.1** | **39.4** | **39.6** | **33.3** | NA | NA | unweighted |
| **Yemen** | **WVS** | **2010/14** | **639** | **39.2 (35.4–43.0)** | **39.1** | **39.4** | **39.6** | **33.3** | **NA** | **NA** | **weighted** |
| Zimbabwe | WVS | 2010/14 | 767 | 41.6 (38.1–45.1) | **40.1** | **42.9** | **47.1** | **48.5** | NA | NA | unweighted |
| **Zimbabwe** | **WVS** | **2010/14** | **767** | **46.7 (42.2–51.3)** | **47.1** | **46.5** | **49.2** | **54.8** | **NA** | **NA** | **weighted** |
| Zimbabwe | WVS | 2017/21 | 790 | 61.7 (58.2–65.1) | 60.7 | 62.6 | 69 | 65.3 | 57.7 | 63.3 | unweighted |
| **Zimbabwe** | **WVS** | **2017/21** | **790** | **61.7 (58.2–65.1)** | **60.7** | **62.6** | **69** | **65.3** | **57.7** | **63.3** | **weighted** |
